# Development and Evaluation of Docetaxel-Loaded Nanostructured Lipid Carriers for Skin Cancer Therapy

**DOI:** 10.3390/pharmaceutics16070960

**Published:** 2024-07-19

**Authors:** Florentina-Iuliana Cocoș, Valentina Anuța, Lăcrămioara Popa, Mihaela Violeta Ghica, Mihaela-Alexandra Nica, Mirela Mihăilă, Radu Claudiu Fierăscu, Bogdan Trică, Cristian Andi Nicolae, Cristina-Elena Dinu-Pîrvu

**Affiliations:** 1 Department of Physical and Colloidal Chemistry, Faculty of Pharmacy, “Carol Davila” University of Medicine and Pharmacy, 6 Traian Vuia Str., 020956 Bucharest, Romania; florentina.cocos@drd.umfcd.ro (F.-I.C.); lacramioara.popa@umfcd.ro (L.P.); mihaela.ghica@umfcd.ro (M.V.G.); mihaela.nica@drd.umfcd.ro (M.-A.N.); cristina.dinu@umfcd.ro (C.-E.D.-P.); 2 Innovative Therapeutic Structures Research and Development Centre (InnoTher), “Carol Davila” University of Medicine and Pharmacy, 6 Traian Vuia Str., 020956 Bucharest, Romania; 3 Center of Immunology, Ștefan S. Nicolau Institute of Virology, Romanian Academy, 030304 Bucharest, Romania; mirela.mihaila@virology.ro; 4 Faculty of Pharmacy, Titu Maiorescu University, 16 Gheorghe Sincai Blvd, 040314 Bucharest, Romania; 5 National Institute for Research & Development in Chemistry and Petrochemistry—ICECHIM Bucharest, 202 Spl. Independentei, 060021 Bucharest, Romania; fierascu.radu@icechim.ro (R.C.F.); trica.bogdan@gmail.com (B.T.); cristian.nicolae@icechim.ro (C.A.N.); 6 Faculty of Chemical Engineering and Biotechnology, National University of Science and Technology Politehnica Bucharest, 1-7 Gh. Polizu Str., 011061 Bucharest, Romania

**Keywords:** docetaxel, anticancer, nanostructured lipid carriers (NLC), lamellar systems, transdermal drug delivery, design of experiments

## Abstract

This study focuses on the design, characterization, and optimization of nanostructured lipid carriers (NLCs) loaded with docetaxel for the treatment of skin cancer. Employing a systematic formulation development process guided by Design of Experiments (DoE) principles, key parameters such as particle size, polydispersity index (PDI), zeta potential, and entrapment efficiency were optimized to ensure the stability and drug-loading efficacy of the NLCs. Combined XRD and cryo-TEM analysis were employed for NLC nanostructure evaluation, confirming the formation of well-defined nanostructures. In vitro kinetics studies demonstrated controlled and sustained docetaxel release over 48 h, emphasizing the potential for prolonged therapeutic effects. Cytotoxicity assays on human umbilical vein endothelial cells (HUVEC) and SK-MEL-24 melanoma cell line revealed enhanced efficacy against cancer cells, with significant selective cytotoxicity and minimal impact on normal cells. This multidimensional approach, encompassing formulation optimization and comprehensive characterization, positions the docetaxel-loaded NLCs as promising candidates for advanced skin cancer therapy. The findings underscore the potential translational impact of these nanocarriers, paving the way for future preclinical investigations and clinical applications in skin cancer treatment.

## 1. Introduction

Recent decades have marked a significant shift in pharmaceutical sciences, primarily driven by the need for more effective drug delivery systems, aiming to enhance therapeutic outcomes, extend the duration of drug release, and improve patient adherence. Among these emerging technologies, nanostructured lipid carriers (NLCs) have garnered attention due to their unique ability to optimize drug delivery through a highly controlled and targeted approach [1].

First introduced in the late 1990s, NLCs evolved from solid lipid nanoparticles (SLNs) by integrating a mixture of solid and liquid lipids in a heat-controlled crystallization process [2,3], presenting an innovative solution to several limitations faced by earlier formulations [4]. Compared to SLNs, NLCs offer a disorganized crystalline matrix with more imperfections in crystal to accommodate the drug, which in turn ensures higher drug loading capacity and minimal drug leakage during storage, addressing key issues of drug stability and release rates [5,6,7].

Numerous preparation techniques are currently used for formulating NLCs, enabling the creation of effective and stable formulations. High-pressure homogenization stands as the most prominent technique due to its ability to consistently produce particles with uniform size and distribution [3,8,9,10,11]. Additional methods include emulsification–ultrasonication, which combines high shear mixing with ultrasonic waves to disperse the lipids [5,12,13], microemulsion technique, which employs both water and oil phases to achieve nano-sized carriers [14,15], the film ultrasonication–dispersion technique, that utilizes ultrasonic energy to disperse a preformed thin lipid film [4], and solvent diffusion technique, where the carrier matrix is formed by the diffusion of a solvent, leading to the encapsulation of the drug [16,17,18]. Recent advancements have led to the development of novel techniques such as those utilizing supercritical fluids [19] and microfluidic platforms [20]. These innovative approaches offer advantages in terms of precise control over particle size, morphology, and enhanced drug encapsulation efficiency.

NLCs can encapsulate a wide range of hydrophobic [5,8,9,10,12] or hydrophilic [13,21] drugs, thus having broad applicability across different therapeutic categories. Additionally, the biocompatible and biodegradable nature of the lipid materials used in formulation ensures minimal in vivo toxicity, a critical consideration for clinical applications [4,10,22]. The surface of NLCs can also be specifically modified to target particular tissues or cellular pathways, enhancing therapeutic efficacy and reducing systemic side effects [23,24,25,26]. 

Currently, NLCs are being explored for their ability to improve the solubility, stability, and bioavailability of poorly water-soluble drugs, as well as to provide controlled and sustained drug release [27,28]. One significant area of focus is the optimization of NLC formulations for improving the systemic bioavailability of poorly soluble drugs, resulting in the formulation of hydrochlorothiazide [2], axitinib [29], carbamazepine [30], atorvastatin [12], or erlotinib [26] NLCs with superior bioavailability to the commercial formulations. 

Since NLCs are particularly advantageous for delivering hydrophobic drugs, which are often used in cancer treatment, they are currently placed in the forefront of innovative cancer treatment strategies, with extensive research and development efforts aimed at enhancing drug delivery, bioavailability, and therapeutic efficacy. This focus is especially relevant for skin cancers (including melanoma and non-melanoma types), which represent a growing global health concern with rising incidence rates [31,32]. Despite advancements in treatment modalities, there remains a critical need for innovative therapeutic approaches with enhanced efficacy and reduced side effects. Nanotechnology-based drug delivery systems, with NLCs as a prime example, offer a promising platform to address this need. By improving drug solubility, bioavailability, ensuring high stratum corneum permeability, and enabling controlled and targeted delivery to the tumor site, while minimizing system toxicity, NLCs have the potential to transform skin cancer treatment and alleviate its global burden [4,10,22,23]. For instance, riluzole-loaded NLCs for topical application have shown potential in treating hyperproliferative skin conditions by inhibiting keratinocyte cell proliferation and providing sustained drug release [33], doxorubicin-loaded NLCs have also shown increased cytotoxicity and drug uptake compared to the plain drug [34], whereas topotecan-loaded NLCs have been found to maintain drug stability and enhance skin permeation, which is crucial for achieving therapeutic drug levels in the skin [35].

One such drug that could benefit significantly from NLC-based delivery is docetaxel, a semi-synthetic taxane second-generation anti-neoplastic agent derived from *Taxus baccata* [36,37,38]. Docetaxel disrupts cancer cell growth by interfering with microtubule polymerization, ultimately triggering apoptosis [37,38,39]. However, its low water solubility (4.93 µg/mL) [36,38,39,40] and high lipophilicity (log*p* value of 4.1) [36,40] pose significant challenges for formulation and administration. Additionally, commercial products containing docetaxel have been associated with several adverse reactions, including severe allergic reactions, systemic toxicity, hypotension, bronchospasm, urticaria, fluid retention, extravasation, and thrombosis [36,37,40,41,42,43].

While docetaxel has shown efficacy in treating a range of malignancies, including breast, lung, and prostate cancers [44,45,46], its clinical utility in skin cancer treatment remains largely unexplored. This is primarily due to two key challenges: the drug’s limited ability to penetrate the skin’s outermost layer, the stratum corneum [47], hindering topical application, and the systemic toxicity associated with conventional docetaxel products, which severely restricts its use for systemic therapy. Advanced lipid nanoparticle systems, such as NLCs and SLNs, offer novel approaches to overcome these limitations by encapsulating docetaxel within a lipid matrix, enhancing its delivery and therapeutic efficacy.

For docetaxel parenteral use, studies have demonstrated that the lipid nanoparticles can improve a drug’s pharmacokinetics and biodistribution, leading to enhanced bioavailability, better tumor targeting, and reduced systemic toxicity [4,8,10,36,48]. Similarly, studies have shown an increased intestinal absorption and lymphatic uptake after oral administration of cysteine-functionalized docetaxel NLCs [40], as well as for surface-modified docetaxel SLNs [37].

While the potential of NLCs and SLNs for systemic docetaxel delivery is well-documented, their application in topical delivery, particularly for skin cancer, remains largely unexplored. This approach holds significant promise as it allows for localized treatment directly at the tumor site, potentially minimizing systemic exposure and side effects, while ensuring sustained drug release. A few recent studies have demonstrated the feasibility of topical docetaxel delivery using lipid nano carriers. One such approach involved development of docetaxel-loaded nano liquid crystals using an emulsification solvent diffusion method, which showed enhanced drug uptake, enhanced drug penetration through the skin, prolonged drug release, and increased cytotoxicity against skin cancer cells compared to the plain drug [49]. Furthermore, de Moura et al. described a hybrid NLC-in-hydrogel system designed for the topical treatment of melanoma, which demonstrated improved drug delivery and therapeutic outcomes [5]. 

Building upon this foundation, our research aims to advance the development of novel NLC formulations for sustained delivery of docetaxel, specifically for topical administration in skin cancer treatment. By optimizing formulation parameters such as lipid composition and drug loading, we aim to tailor optimized NLCs able to achieve sustained drug release, prolonged retention at the tumor site, and enhanced intracellular uptake within cancer cells, ultimately aiming to bridge the gap between promising preclinical results and clinical translation for topical nanomedicine in skin cancer treatment. 

By employing the principles of Quality by Design, we aim to meticulously tailor and improve the properties of these carriers. To achieve this, we will utilize experimental designs, notably the Design of Experiments (DoE) methodology, facilitating a systematic approach towards formulation development and optimization.

## 2. Materials and Methods

### 2.1. Materials

Docetaxel (ScinoPharm, Tainan, Taiwan), Lipoid S-PC-3 (Lipoid GmbH, Ludwigshafen, Germany), Gelucire^®^ 43/01 (Gattefosse, Saint-Priest, France), glyceryl caprylate (Ellemental, Oradea, Romania), Miglyol 812 (MCT) (IOI Oleo GmbH, Witten, Germany), and Tween 80 (Scharlau, Barcelona, Spain) were used for the preparation of NLCs. Additional lipids and surfactants included stearic acid, palmitic acid, cetyl palmitate, alpha tocopheryl acetate, retinyl palmitate, oleic acid, DL-limonene, 1,2 propanediol, and isopropyl myristate (all purchased from Merck, Darmstadt, Germany); decanoic acid, sorbitan monostearate, tetradecanoic acid, sodium taurocholate, sodium deoxycholate, sodium cholate, Brij^®^ 35, Triton^®^ X 100, Tween 60, Tween 20, and Span 80 (obtained from Sigma Aldrich, Saint Louis, MO, USA), Gelucire^®^ 50/13, Gelucire^®^ 48/16, Gelucire^®^ 44/14, Suppocire^®^ NAS 50, and Transcutol^®^ (Gattefosse, Saint-Priest, France); Lipoid E80, Phospholipon^®^ 90G, Lipoid PG 18:0/18:0, and Lipoid E PC S (Lipoid GmbH, Ludwigshafen, Germany). Kolliphor^®^ P188, Kolliphor^®^ P407, and Kolliphor^®^ EL were obtained from BASF, Ludwigshafen am Rhein, Germany. Glyceryl monostearate (GMS) was obtained from Cognis GmbH, Monheim am Rhein, Germany, whereas self-emulsifying glyceryl monostearate (GMS-SE) was purchased from Ellemental, Oradea, Romania. Oat oil, Neem oil, Pomegranate oil, Sesame oil, Grape seed oil, Jojoba oil, and Wheat germ oil were obtained from various commercial sources.

HPLC gradient grade acetonitrile and methanol were procured from Honeywell Research Chemicals, Seelze, Germany. LC-MS grade formic acid was obtained from Fisher Chemical, Pardubice, Czech Republic. Potassium phosphate monobasic, disodium hydrogen phosphate, sodium chloride, and ethanol for analysis were acquired from Merck, Darmstadt, Germany, while potassium chloride was sourced from Lach:ner, Germany. Ultrapure water (resistivity of 18.2 MΩ·cm at 25 °C and a total organic carbon (TOC) content below 5 ppb), was produced using a Milli-Q EQ 7008 water purification system (Merck Millipore, Burlington, MA, USA).

Finally, 5-Fluorouracyl (5-FU), PBS/1 mM EDTA, L-Glutamine (Glu), Penicillin (100 units/mL), Streptomycin (100 μg/mL), Dulbecco’s modified Eagle’s medium (DMEM), fetal bovine serum (FBS), Propidium Iodide (PI) (stock solution 4 mg/mL PI in PBS), and RNase A (stock solution 10 mg/mL RNase A) were purchased from Sigma Aldrich (St. Louis, MO, USA). Annexin V-FITC kit and CycleTEST PLUS DNA Reagent kit were purchased from Becton Dickinson Biosciences (San Jose, CA, USA).

### 2.2. Selection of Lipids and Surfactants

A preliminary excipient screening based on the equilibrium solubility of docetaxel in various solid and liquid lipids and several surfactants was performed to select the optimal NLC components.

#### 2.2.1. Selection of the Liquid Lipids

To ascertain the solubility of docetaxel, multiple liquid lipids (oleic acid, isopropyl myristate, 1,2—propanediol, DL-limonene, glyceryl caprylate, Miglyol^®^ 812N, Retinyl palmitate, α-Tocopheryl acetate, Oat oil, Neem oil, Pomegranate oil, Sesame oil, Grape seed oil, Jojoba oil, and Wheat germ oil) were evaluated. Excess docetaxel was added to 350 mg of accurately weighted liquid lipids in 2 mL polypropylene test tubes. This step was followed by vigorous mixing using a vortex mixer for 5 min to homogenize the sample, facilitating better interaction between the lipid and docetaxel. The quantity of the active pharmaceutical ingredient (API) was incrementally augmented if needed, to ensure a supersaturated system [10,14,50,51,52,53]. The mixture of drug and lipid was maintained at 32 °C and 750 rpm using a ThermoMixer C system fitted with an appropriate size thermo-block (Eppendorf, Hamburg, Germany) for 24 h. The samples were further centrifuged at 15,000 rpm by an SL1R Plus centrifuge (Thermo Fisher Scientific, Karlsruhe, Germany), followed by carefully withdrawing a sample of the supernatant from each vial without disturbing the undissolved drug particles. After appropriate dilution, the samples were analyzed by HPLC to quantify the amount of docetaxel dissolved in each solvent.

#### 2.2.2. Selection of the Solid Lipids

To assess the solubility of the active substance, various solid lipids were examined (such as tetradecanoic acid, decanoic acid, palmitic acid, stearic acid, GMS-SE, GMS, sorbitan monostearate, cetyl palmitate, Gelucire^®^ 43/01, and Suppocire^®^ NAS 50). Initially, 500 mg of each solid lipid was accurately weighed and melted. Subsequently, excess docetaxel was added to the molten lipid. The drug–lipid mixture was kept at a constant temperature of 75 °C, thus maintaining a minimum of 10 °C above the melting point of the solid lipids. The mixture was stirred at 750 rpm for 24 h. Using preheated utensils, a predetermined amount of supernatant was carefully transferred into a 5 mL volumetric flask. The transfer was done by weight to ensure precision and consistency in sample size across all tests. The transferred sample was dissolved using a suitable solvent selected based on its compatibility with both the lipid and docetaxel. Choices like methanol, dichloromethane, DMSO, or their mixtures were used depending on their efficacy in dissolving the sample thoroughly. After dissolution, the sample was further diluted to achieve the desired concentration for HPLC analysis as well as compatibility with the mobile phase. 

#### 2.2.3. Selection of Surfactants

Various surface active agents, such as Tween 80, Tween 60, Tween 80, Lipoid E80, Phospholipon^®^ 90G, Lipoid PG 18:0/18:0, Lipoid E PC S, Lipoid S-PC-3, Kolliphor^®^ EL, Triton^®^ X-100, Brij^®^ 35, sodium deoxycholate, sodium cholate, sodium taurocholate, Transcutol^®^, Kolliphor^®^ P 88, Span 80, Gelucire^®^ 50/13, Gelucire^®^ 48/16, and Gelucire^®^ 44/14 were evaluated both in terms of drug solubilization capacity and in that of NLC stabilization. Their capacity for docetaxel solubilization was assessed differently, based on their different properties. Docetaxel equilibrium solubility was evaluated either for the surfactant dispersed in water (1% *w*/*w*) or in the pure surfactant at 32 °C if liquid and at 75 °C if solid at room temperature. 

#### 2.2.4. Docetaxel Quantitative Analysis

For the quantitative analysis of docetaxel, a Jasco LC-4000 Series HPLC system (JASCO Corporation, Tokyo, Japan) containing a quaternary pump (model PU-4180), an autosampler (model AS-4150), a DAD detector (model MD-4010), and a column thermostat (model CO-4061) was employed. The separation was conducted at λ = 230 nm using a 100 × 3 mm and 2.6 µm particle size Kinetex^®^ C18 column, maintained at 45 °C (Phenomenex, Torrance, CA, USA). The mobile phase was composed of 0.1% formic acid (mobile phase A) and a 1:1 (*v*:*v*) mixture of acetonitrile and methanol (mobile phase B). The gradient elution commenced with a 45:55 (*v*/*v*) ratio of mobile phase A to mobile phase B, maintained for 6 min, followed by a system wash using 95% mobile phase B to ensure the complete removal of any residual fatty acids.

The assay method used for quantifying docetaxel was validated according to the ICH guidelines [54] to ensure its linearity, accuracy, precision, specificity, and sensitivity. 

### 2.3. Preparation of Docetaxel-Loaded NLCs

**Figure 1:**
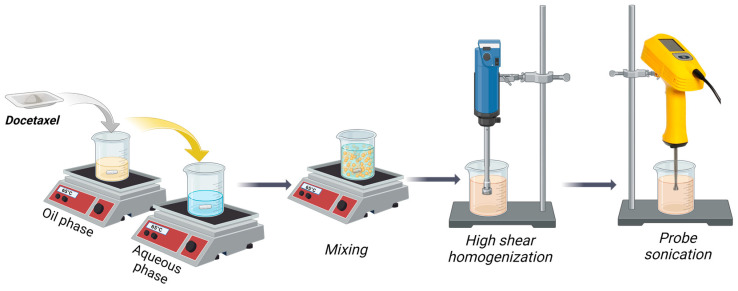
Schematic representation of NLC preparation process (created with BioRender.com).

NLCs were prepared using a modified emulsification–ultrasonication method [5,55], depicted schematically in Figure 1. 

Briefly, Gelucire^®^ 43/01 (G43/01) was melted at 65 °C on a Hei-PLATE Mix’n’Heat Expert magnetic hot-plate stirrer (Heidolph Instruments GmbH & Co. KG, Schwabach, Germany). Glyceryl caprylate, MCT, phosphatidylcholine, and docetaxel were then dissolved sequentially into the molten lipid phase. An aqueous phase containing Tween 80 was preheated to 65 °C, and the lipid phase was quantitatively added and maintained under continuous stirring for 10 min. The resulting mixture underwent a two-step homogenization using an Ultra-Turrax^®^ T25 digital high-shear homogenizer (IKA-Werke GmbH & Co. KG, Staufen im Breisgau, Germany): initial mixing at 3000 rpm for 5 min, followed by further homogenization at 15,000 rpm for 10 min. The pre-emulsion formed was further sonicated using a UP200Ht probe-tip sonicator (Hielscher Ultrasonics GmbH, Teltow, Germany) at 50 W power and 50% duty cycle (alternating 30 s on/off cycles) for 15 min.

For long-term preservation, the prepared NLCs were freeze-dried by using 5% mannitol as a cryoprotectant. For comparison, a blank NLC formulation, devoid of docetaxel, was similarly prepared.

### 2.4. Experimental Design

A three-factor, three-level Box–Behnken experimental design, generated using Design Expert version 13 software (Stat-Ease Inc., Minneapolis, MN, USA), was employed to optimize the formulation of the docetaxel NLCs. The independent variables, outlined in Table 1, included the percentage of liquid lipids in the oil phase (LL%), the concentration of Tween 80 in the aqueous phase (T80%), and the docetaxel loading (DTX, expressed as mg docetaxel per gram of lipids). Nanoparticle characteristics, including particle size immediately after preparation (Size) and after 30 days of storage (Size SS), polydispersity index (PDI), zeta potential (ZP), and cumulative amount of docetaxel released after 24 h (Release 24 h), served as the optimization criteria. A constant lipid content of 5% *w*/*w* was maintained, with phosphatidylcholine representing 10% (*w*/*w*) of the oil phase. The two liquid lipids were incorporated at a 1:1 weight ratio.

The design matrix comprised 15 analytical experiments (Table 2), employing a randomized run order to mitigate potential bias from uncontrolled variables. Three replicates of the central point were included, in order to enhance the model’s ability to detect curvature in the response surface and to provide a robust estimate of experimental error.

To investigate the relationship between the measured response and independent variables, a second-order polynomial equation was developed using multiple regression analysis:

$$Y=b_{0}+\sum_{i=0}^{n}b_{i}X_{i}+\sum_{i=0}^{n}b_{ii}X_{i}^{2}+\sum_{i<j}^{n}b_{ij}X_{i}X_{j}+\varepsilon$$

In the equation, Y represents the measured response, b denotes the coefficients calculated by multiple regression analysis, $X_{i}$ represent the main effects of the independent variables, $X_{i}X_{j}\;$ are the interaction terms between variables, $X_{i}^{2}$ represents the quadratic expressions of the independent variables (included to investigate nonlinearity), and ε is the random error, accounting for any unexplained variation in the response. The quadratic models developed were subsequently refined using backward elimination based on the Akaike criterion, removing non-significant terms with a *p*-value greater than 0.05 [56].

Analysis of variance was used to assess the significance of the regression model and identify key experimental factors (*p*-value < 0.05). The F-test and *p*-value assessed overall significance, while the coefficient of determination (R²) measured the proportion of response variation explained by the model. Response surface methodology, including contour plots, surface response plots, and interaction plots, was employed to visually interpret and explain the relationships captured by the models.

### 2.5. NLC Characterization

#### 2.5.1. Nanoparticle Size and Polydispersity Index (PDI) by Dynamic Light Scattering (DLS)

The analysis of the average hydrodynamic diameter and PDI of nanoparticles was performed through the DLS technique. The samples were diluted (200×) in the aqueous phase of each formulation to obtain a suitable scattering intensity. Precise measurements were conducted at room temperature employing a VascoKin particle analyzer (Cordouan Technologies, Pessac, France) featuring a 638-nanometer laser. Additionally, particle behaviour was investigated utilizing an in situ probe operating in back-scattering mode with a scattering angle of 170°. Calibration was executed by using a standardized colloidal dispersion of 1% latex containing 100 nm sized particles. The size distribution (nm) based on intensity (a.u.) was assessed for each NLC sample using the Cumulant algorithm, which is correlated with the autocorrelation function expressed in time (µs) relative to intensity (a.u.). The resulting data were fitted using the Rayleigh model [57,58,59,60]. The samples were analyzed immediately after preparation and after 30 days (for stability evaluation).

#### 2.5.2. Zeta Potential Determination

The analysis of zeta potential was carried out through the application of the laser Doppler electrophoresis technique [61]. The Wallis Zeta potential analyzer (Cordouan Technologies, Pessac, France) was employed for measurements, incorporating a 20 mW diode laser functioning at a wavelength of 635 nm. For equipment calibration, a 0.5% Ludox TM-50 colloidal silica standard (Sigma Aldrich, Saint Louis, MO, USA) was used. The NLC samples were diluted (400×) in double distilled water for this analysis. The outcomes presented represents the mean values acquired from ten individual determinations [5,60].

#### 2.5.3. Entrapment Efficiency

The encapsulation efficiency (EE%) of docetaxel was determined by the ultrafiltration–centrifugation method [55,62,63], using polyethersulfone filters 100,000 MWCO (Vivapin^®^ 500, Sartorius, Epsom, UK). Each sample was diluted 50 times in 0.5% wt% Tween 80 phosphate buffer solution (PBS), pH = 7.4 [4]. 100 µL of the obtained dispersion was subjected to ultracentrifugation at 15,000 rpm for 10 min to separate free docetaxel. Subsequently, 100 µL methanol was added five times into the ultracentrifugation filter to dissolve the entrapped API. All collected methanolic fractions were diluted with an equal volume of water:methanol = 1:1 (*v*/*v*) mixture and then analyzed by the HPLC method described above. The resulting outcome was utilized for the calculation of the encapsulation efficiency using the formula below:

(1) $$EE\left(\%\right)=\frac{Quantity\;of\;DTX\;in\;NLC}{Total\;amount\;of\;DTX}\times 100$$

#### 2.5.4. In Vitro Release of NLC-DTX 

An in vitro release study of docetaxel was conducted using a modified dialysis technique [64,65]. The experiment employed a Sotax CE7 smart USP Apparatus 4 integrated with a CP7 piston pump (Sotax AG, Aesch, Switzerland) equipped with 22.6 mm flow cells, operated under a closed loop regimen. Each cell was prepared by positioning a 5 mm ruby bead at the apex of the cone to safeguard the inlet tube. Additionally, approximately 8.0 g of 1 mm glass beads were placed in the cone area to maintain a uniform flow of the medium across the sample, preventing channeling and ensuring consistent contact with the sample. A volume of 1.2 mL of each NLC formulation was introduced into a preconditioned SpectraPor^®^ Float-A-Lyzer^®^ G2 Dialysis Device with a 100 kD MWCO (Repligen, Waltham, MA, USA), which was then fitted into the flow cell using specialized dialysis cap adapters (Sotax AG, Aesch, Switzerland). The system circulated in a closed loop 250 mL of PBS at pH 7.4 containing 20% ethanol (in order to maintain sink conditions) at a constant flow rate of 8 mL/min and 37 °C. At designated intervals, 1 mL of the medium was withdrawn and promptly replaced with an equivalent volume of fresh preheated medium to preserve a constant volume [65]. All collected samples were analyzed via HPLC. 

In the drug release study, 20% ethanol was used in the dissolution medium to enhance the solubility of docetaxel and maintain sink conditions. This choice was based on preliminary solubility tests, which confirmed that the medium could fully dissolve the released docetaxel, ensuring that the cumulative percentage of released drug was accurately measured.

#### 2.5.5. Scanning Electron Microscopy (SEM)

For SEM sample preparation, a droplet of each NLC suspension was placed on a conductive carbon tape (TED PELLA Inc., Redding, CA, USA) securely attached to an aluminum SEM stub. The samples were sputter-coated using a gold–palladium target under an argon gas atmosphere to ensure electrical conductivity and compatibility with scanning electron microscopy analysis. The coating was performed for 1 min, with a plasma current of 25 mA, using a SC7620 Mini Sputter Coater (Quorum Technologies, Laughton, UK). Samples were stored in a desiccator until analysis. The samples were inspected using a TM4000Plus scanning electron microscope (Hitachi, Tokyo, Japan), operated at a voltage setting of 15 kV, under backscattered electron detection (BSE) mode.

#### 2.5.6. Cryo-Transmission Electron Microscopy (Cryo-TEM)

The samples were vitrified using the Vitrobot Mark IV (Thermo Fischer Scientific, Waltham, MA, USA). The blotting process occurred at 4 °C at a relative humidity of 100% with 3 consecutive blotting operations each lasting 3 s with a force value of 3. 10 µL of sample were applied onto an S166 lacey carbon film 200-mesh copper grid (Agar Scientific, Stansted, UK). After blotting, the grid was plunged into cooled liquid ethane. Afterwards, it was carefully moved to the cryo-holder (model 626, Gatan, Pleasanton, CA, USA). Samples were analyzed at an accelerating voltage of 120 kV on the Tecnai F20 G² TWIN Cryo-TEM (Thermo Fischer Scientific, Waltham, MA, USA). 

#### 2.5.7. X-ray Diffraction

X-ray diffraction (XRD) studies were performed using a Rigaku SmartLab 9 kW equipment, in 2ϴ/ϴ configuration, operating at 45 kV and 200 mA, using Cu_Kα_ radiation—1.54059 Å), between 2 and 90° (2θ). The analysis of the registered diffractogram was performed using the PDXL 2.7.2.0. software (Rigaku Corporation, Tokyo, Japan), while the identification of the individual components was performed by comparison with ICDD data entries or using the available literature data. 

The interplanar spacing (d-spacing), i.e., the distance between adjacent planes of atoms within a regular, repeating crystal lattice, was calculated using Bragg’s Law:

nλ=2dsin⁡θ

where n is the order of reflection (1, 2, 3…), λ is the X-ray wavelength, d = interplanar spacing, and θ is the Bragg angle.

#### 2.5.8. Thermal Analysis

Thermogravimetric analysis (TGA) was performed for NLC-DTX, NLC-Blank, and their components (DTX, GC, G43/01, MCT, S-PC-3, T80, mannitol) using thermal analyzer TGA Q5000IR (TA Instruments, New Castle, DE, USA). TGA curves were generated with a heating rate of 10 °C/min over a temperature range from room temperature to 700 °C, under a dynamic nitrogen atmosphere (50 mL/min). The samples were placed in a platinum pan (100 µL) [66,67,68]. 

### 2.6. In Vitro Biological Testing 

#### 2.6.1. Cell Culture and Treatments 

SK-MEL-24 (human melanoma cell line—HTB-71) and HUVEC (human primary umbilical vein endothelial cells—CRL-1730) were purchased from the American Type Culture Collection (ATCC). HUVEC normal cells were used as a reference. Adherent cells were maintained in culture in DMEM/F12 medium added by 2 mM L-glutamine, 10% fetal bovine serum, 100 units/mL penicillin, 100 μg/mL streptomycin, and incubated at 37 °C in 5% CO_2_ humidified atmosphere. After 24 h, adherent cells were treated with different concentrations of the compounds for different timeframes. A 5-FU conventional oncology drug used in cancer treatments can be used as a positive control of the experiments. Cell treatments of compounds were carried out using concentrations of 200, 100, 50, 25, 12.5, 6.25, 3.125, and 1.56 μg/mL of the drug. Then, cells were detached with a nonenzymatic PBS/1 mM EDTA solution washed twice in PBS.

#### 2.6.2. Cytotoxicity Assay

All assays were performed in triplicate in 96-well microtiter plates with flat bottom (Falcon), using CellTiter 96 Aqueous One Solution Cell Proliferation Assay (Promega, Madison, WI, USA), an MTS-based colorimetric assay. The method is based on the ability of metabolically active cells to reduce MTS, a yellow tetrazolium salt, to the colored formazan that is soluble in the culture medium. The mixture reagent used consists of MTS, [3-(4,5-dimethylthiazol-2-yl)-5-(3-carboxymethoxy-phenyl)-2-(4-sulfophenyl)-2H-tetrazolium, inner salt], and PES (phenazine ethosulfate), that has high chemical stability and might be combined with MTS to form a stable solution. Then, 1 × 10^4^ cells/well were cultured in 100 μL for 24 h, culture supernatants were discarded, then normal and cancer cells were treated for 24 and 48 h with increasing concentrations of compounds or oncolytic drug. After the end of the incubation time, 20 μL reagent containing a) MTS, (3-(4,5-dimethylthiazol-2-yl)-5-(3-carboxymethoxyphenyl)-2-(4-sulfophenyl)-2H-tetrazolium, inner salt) and b) PES (phenazine methosulfate), were added in each well. Plates were incubated for 4 h at 37 °C, with mild agitation every 15 min. The reduction of the tetrazolium compound to formazan was spectrophotometrically measured at λ = 492 nm using a Dynex plate reader (DYNEX Technologies MRS, Chantilly, VA, USA). The percentage of viability compared to untreated cells (considered 100% viable) was calculated with the formula:

(2) $$Cell\;viability=\frac{absorbance\;of\;treated\;cells-absorbance\;of\;culture\;medium}{absorbance\;of\;untreated\;cells-absorbance\;of\;culture\;medium}\times 100$$

The percentage of viability compared to untreated cells was calculated, and data were expressed as mean ± standard deviation (SD) of the experiments, obtained in triplicate (*n* = 3) [69,70].

#### 2.6.3. Apoptosis Analysis

The apoptosis assay was performed according to the manufacturer’s instructions using the Annexin V-FITC kit from BD Biosciences. Cells treated and untreated with the experimental compound were resuspended in a cold binding buffer. The percentages of apoptotic cells were determined by double staining with Annexin V-FITC/PI. In each tube was added 400 μL of Annexin V binding buffer and the 5000 cells/sample for 15 min in the dark at room temperature. The cells were analyzed using a FACS CantoII flow-cytometer (Becton Dickinson—BD). The analysis was performed using DIVA 6.2 software to discriminate viable cells (FITC-PI-) from necrotic cells (FITC+PI+) and early apoptosis (FITC+PI-) from late apoptosis [71,72].

#### 2.6.4. Cell Cycle Analysis

In this experiment, 5 × 10^5^ cells that had been previously fixed were washed and resuspended in PBS. The CycleTEST PLUS DNA Reagent kit from BD Biosciences was used according to the manufacturer’s protocol for the assay. The probes were kept in the dark and at 4 °C until data acquisition by flow cytometry (Becton Dickinson—BD) using a FACSCantoII flow cytometer from BD. The ModFIT v3.2 software was used to analyze the data and estimate the DNA index (DI) and progression through cell cycle phases [73].

## 3. Results and Discussion

### 3.1. Docetaxel Quantitative Analysis

**Figure 2:**
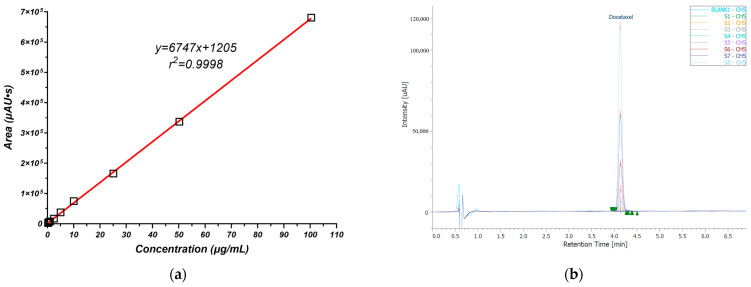
(**a**) Calibration curve for docetaxel in the range of 0.25–100 μg/mL. (**b**) Overlaid chromatograms of docetaxel standards and a matrix blank, demonstrating the sensitivity and selectivity of the method for quantifying docetaxel.

Docetaxel quantification method exhibited excellent linearity (R² = 0.9998) over a concentration range of 0.25–100 µg/mL, with a calibration curve defined by the equation y = 6747x + 1205 (Figure 2a). All analyses were performed in triplicate. Assay specificity, assessed by evaluating potential interferences from drug-free media components, demonstrated the method’s ability to accurately quantify docetaxel in the presence of excipients. The quantitation limit (LOQ), determined using a 10:10 signal-to-noise ratio, was evaluated at 0.062 μg/mL, further establishing the method’s sensitivity for docetaxel analysis.

### 3.2. Selection of Lipids and Surfactants

**Figure 3:**
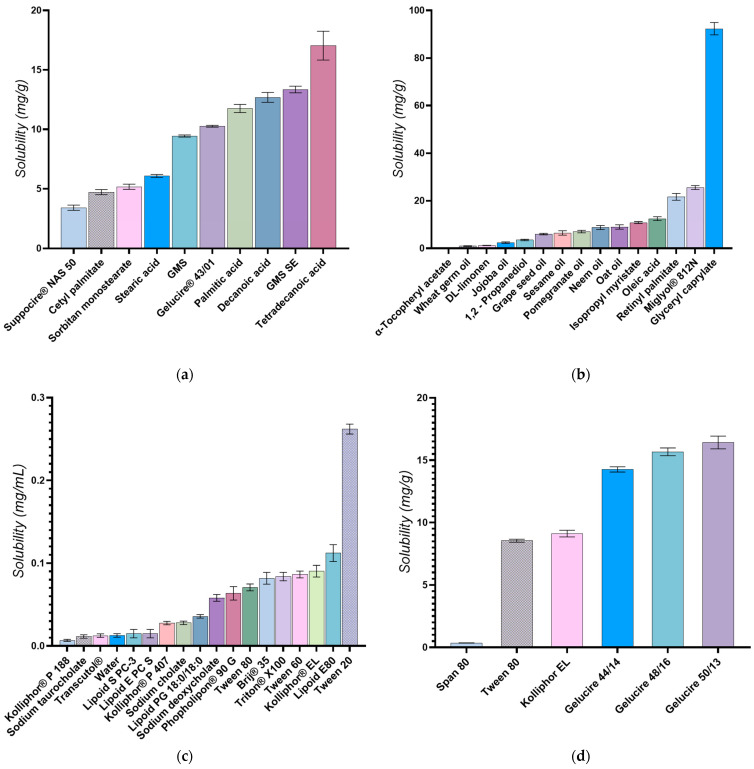
Docetaxel solubility in (**a**) solid lipids in melted state at 75 °C, (**b**) liquid lipids at 32 °C, (**c**) 1% *w*/*w* surfactant aqueous solutions/dispersions, and (**d**) pure surfactants.

The primary criterion for selecting lipids was their ability to solubilize docetaxel effectively. This was crucial because docetaxel’s low water solubility and high lipophilicity pose significant challenges for its formulation [36,40]. Accordingly, a range of solid and liquid lipids were evaluated for their solubilization potential. The results of these solubility studies, depicted in Figure 3a,b, demonstrated variable solubilization capacities across different lipid types. In solid lipids, the highest solubilization capacity for the API was observed with tetradecanoic acid, followed sequentially by decanoic acid, palmitic acid, and stearic acid. Among solid-state esters, the greatest solubility of docetaxel was found in self-emulsifying glyceryl monostearate (GMS-SE), succeeded by Gelucire^®^ 43/01, standard glyceryl monostearate (GMS), sorbitan monostearate, cetyl palmitate, and Suppocire^®^ NAS 50. While tetradecanoic acid demonstrated the highest solubility (16 mg/g), Gelucire^®^ 43/01 was ultimately selected as the preferred lipid carrier despite its lower docetaxel solubility (10 mg/g). This decision was driven by a multifaceted evaluation that considered not only drug solubility but also other critical quality attributes pertinent to the intended topical application in skin cancer.

Gelucire^®^, a family of polyethylene glycol esters of fatty acids, is renowned for its biocompatibility, versatility, safety, and wide acceptance as a pharmaceutical excipient, particularly in topical and oral formulations [74]. Importantly, Gelucire^®^43/01 is known to form stable NLCs with desirable controlled release properties [75]. This aligns perfectly with the overarching goal of this study to develop a formulation capable of sustained docetaxel delivery to the tumor site, potentially improving therapeutic efficacy while minimizing systemic exposure and off-target effects.

While several fatty acids exhibited superior docetaxel solubility, their potential advantages were outweighed by the established benefits of Gelucire^®^43/01 in terms of formulation stability, controlled drug release, and biocompatibility. Furthermore, during the preliminary screening phase, NLCs prepared using fatty acids as solid lipids resulted in formulations with large particle sizes and exhibited a loss in stability after just several days. Additionally, Gelucire^®^43/01 presents favorable processing characteristics versus the other solid lipids tested, such as low melting point and ease of handling, which facilitates manufacturing and further supports its selection in the final optimization design.

In terms of liquid lipids, esters derived from free fatty acids, such as glyceryl caprylate, medium-chain triglycerides (MCT/Miglyol^®^ 812N), and vitamin A (retinyl palmitate), demonstrated the highest solubilization capacities for docetaxel. Additionally, oleic acid was notably effective in dissolving docetaxel.

For the preparation of NLCs, the solubility of the API in the lipid matrix is more important than its solubility in the surfactant or surfactant blends. The API needs to be sufficiently soluble in the lipid phase because the primary role of the lipids in NLCs is to encapsulate the API and control its release. A good solubility of the API in the lipid matrix ensures that a higher drug load can be achieved and that the API is uniformly distributed within the lipid matrix. Surfactants in the context of NLC preparation primarily function to stabilize the emulsion and prevent the aggregation of lipid nanoparticles. While it is good for the API to have some level of solubility in the surfactant blends to ensure proper interface stabilization, excessive solubility could potentially lead to the API preferentially partitioning into the surfactant layer, which could affect the drug loading efficiency and release profile. Ultimately, the surfactant is selected based on its ability to form stable nanoparticle dispersions rather than its capacity to solubilize the API. To achieve optimal nanoparticle stabilization, enhanced entrapment efficiency, and robust interactions between the lipid and the drug, emphasis was placed on selecting surfactants with limited docetaxel solubility. The results of these evaluations, illustrated in Figure 3c, indicate that Kolliphor^®^ P188 had the least capacity to solubilize the API, followed by sodium taurocholate, Transcutol^®^, Lipoid S-PC-3, and Lipoid E-PC-S. Furthermore, as shown in Figure 3d, among the surfactants tested independently, Span 80 exhibited the lowest solubilization effect on docetaxel, followed by Tween 80, Kolliphor EL, and the various surfactants from the Gelucire^®^ family.

Based on the solubility study together with the preliminary stability assays, a combination of Lipoid S-PC-3 (a phosphatidylcholine) and Tween 80 was employed to stabilize the NLCs. This decision was based on the distinct properties of each surfactant and their known synergistic interactions [76]. Lipoid S-PC-3, a phospholipid, forms strong, ordered films at the lipid–water interface. Tween 80, a non-ionic surfactant, is a versatile non-ionic surfactant known for its solubilizing, stabilizing, and permeation-enhancing properties, further reduces interfacial tension and can intercalate within the phospholipid layer. This synergistic interaction creates a more robust and tightly packed interfacial film, leading to smaller, more stable NLCs with reduced aggregation tendencies [77]. 

### 3.3. Experimental Design

Based on the solubility studies and preliminary evaluations of their ability to form stable nanostructured systems, the chosen lipid matrix for the nanoparticles comprises Gelucire^®^ 43/01, medium-chain triglycerides (MCT), and glyceryl caprylate. For the formulation, the water-miscible surfactant Tween 80 and the lipid-miscible surfactant Lipoid S-PC-3 were selected. To determine the optimal concentrations of these excipients, a Box–Behnken design was applied to the variables: percentage of liquid lipids in the oil phase (LL, % *w*/*w*), Tween 80 concentration (T80%), and docetaxel loading (DTX, mg/g). The results from the 15 formulations assessed are summarized in Table 2, detailing the properties of interest such as particle size (Size, nm), polydispersity index (PDI), zeta potential (ZP, mV), particle size after 30 days (Size SS, nm), and drug release after 24 h (Release 24h, %). The entrapment efficiency was also evaluated for all the formulations but was not selected as response in the final experiment due to all formulations presenting EE% of more than 95%.

The initial quadratic models were fitted to the data, and backward elimination based on the Akaike criterion was employed to remove non-significant terms (*p*-value > 0.05), ensuring the final models included only significant factors for accurate prediction and optimization of the response variables. Analysis of variance (ANOVA) was then used to assess the significance of the refined regression model and identify key experimental factors (*p*-value < 0.05). The F-test and *p*-value assessed overall significance, while the coefficient of determination (R²) measured the proportion of response variation explained by the model. In Table 3, the resulting models for each response are presented, along with the significant factors influencing the responses and their corresponding *p*-values. The adjusted R² values ranged from 0.8680 to 0.9329 for all fitted models, indicating a high proportion of response variation explained by the models.

The regression equations derived from the experimental data highlight the significant terms influencing each response variable, along with their signs, which indicate the nature of the effect (positive or negative). A positive coefficient suggests that an increase in the corresponding factor leads to an increase in the response, while a negative coefficient indicates a decrease in the response. The obtained equations are:

Size (nm) = 193.3 + 21.81*A − 67.36*B + 48.74*B^2^

PDI = 0.2146 + 0.0221*B + 0.0033*C + −0.0509*BC + 0.0726*C^2^

ZP (mV) = −24.15 + 2.64*A + 2.74*B + 2.95*C + 2.80*C^2^

Size SS (nm) =283.09 − 45.61*A + 38.71*C − 44.23*AC

Released 24 h (%) =36.82 − 0.56*A + 4.43*B − 5.14*C + 5.57*BC + 5.06*A^2^

In the particle size equation, the positive coefficient for liquid lipid load (21.81) suggests that increasing lipid load increases particle size, whereas the negative coefficient for Tween 80 concentration (−67.36) indicates that increasing Tween 80 reduces particle size. Similarly, in the zeta potential equation, the positive coefficients for liquid lipid load, Tween 80, and docetaxel amount (+2.64, +2.74, and +2.95, respectively) demonstrate that all these factors contribute to an increase in zeta potential. The interaction and quadratic terms further refine the model, capturing the complex relationships between variables. For example, the negative coefficient for the interaction between Tween 80 and docetaxel in the PDI equation (−0.0509) signifies that their combined effect reduces PDI, promoting a more uniform particle size distribution.

**Figure 4:**
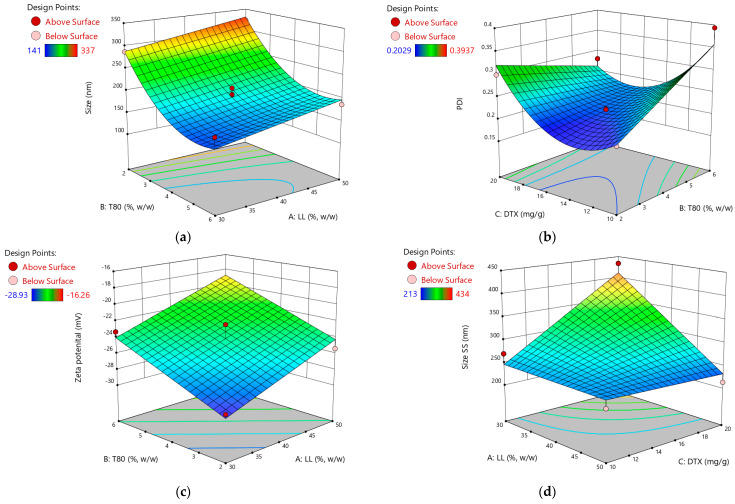

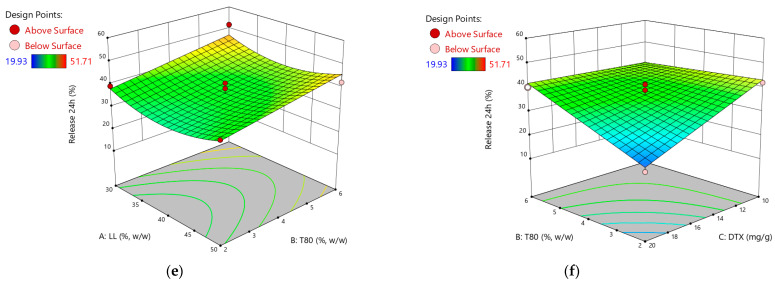
Box–Behnken design results for NLC-DTX system: response surface for size (**a**), PDI (**b**), zeta potential (**c**), size SS (**d**), and Release 24h (**e**,**f**).

Surface graphs illustrating the influence of the statistically significant variables on the responses are presented in Figure 4a–f. 

Particle size is a critical parameter that significantly influences the performance of nanostructured lipid carriers (NLCs) in drug delivery systems. Smaller particle sizes generally enhance the bioavailability of the encapsulated drug due to increased surface area, facilitating more efficient cellular uptake and improved dissolution rates. Additionally, particle size affects the stability, release kinetics, and biodistribution of the NLCs, which are essential factors for effective therapeutic outcomes [78,79]. 

The initial particle size of the NLC formulations ranged from 141 to 337 nm, demonstrating successful fabrication of nanoscale drug delivery systems. The quadratic model fitting the particle size data highlights the significant influence of both the liquid lipid and surfactant components on NLC dimensions. As evidenced by the positive coefficient associated with factor A, increasing the proportion of liquid lipids leads to a predictable increase in particle size. This observation aligns with previous reports [29], suggesting that higher concentrations of liquid lipids within the solid matrix can enhance drug loading capacity but may also result in larger particles. Conversely, the negative linear coefficient for factor B, representing Tween 80 concentration, indicates an inverse relationship with particle size. This finding supports the well-established role of surfactants in reducing interfacial tension, thereby facilitating smaller droplet formation [80]. The model also reveals a significant quadratic effect for Tween 80 (B²), suggesting that at higher concentrations, the surfactant’s impact on particle size reduction plateaus or even reverses. This phenomenon could be attributed to micelle saturation or changes in the interfacial film curvature at elevated surfactant levels [81]. Notably, the amount of docetaxel (factor C) did not significantly influence particle size, indicating that drug loading within the investigated range did not disrupt the NLC self-assembly process or substantially alter the particle dimensions. This finding suggests that the developed formulation strategy allows for efficient drug encapsulation without compromising the desired nanoscale characteristics of the delivery system.

Intriguingly, while the initial particle size was predominantly dictated by the liquid lipid and surfactant concentrations, the particle size at 30 days (Size SS) reveals a shift in influential factors. The negative coefficient for factor A in the equation suggests that higher initial liquid lipid concentrations, while initially leading to larger particle sizes, may contribute to a decrease in size over time. The positive coefficient for the C factor, docetaxel, implies that increasing the docetaxel concentration leads to an increase in particle size after 30 days. This effect might be due to the crystallization or aggregation of docetaxel within the lipid matrix over time, causing an increase in particle size.

The interaction term (−44.23*AC) is negative, indicating a synergistic effect between the liquid lipids and docetaxel that reduces particle size when both are increased. This interaction suggests that while docetaxel alone may contribute to particle growth, its combination with MCT can counteract this effect, leading to smaller and more stable particles over time.

The polydispersity index, ranging from 0.2041 to 0.3937 (Table 2), indicates a relatively narrow size distribution for the formulated NLCs [78,82]. The regression model for PDI highlights the significant interaction between surfactant (B) and drug (C), suggesting a combined effect on particle homogeneity.

The zeta potential values, ranging from −16 to −29 mV, indicate good physical stability of the NLC formulation. The negative ZP values are attributed to the anionic nature of the Tween 80 surfactant stabilizing the nanoparticle surface [83,84,85]. 

**Figure 5:**
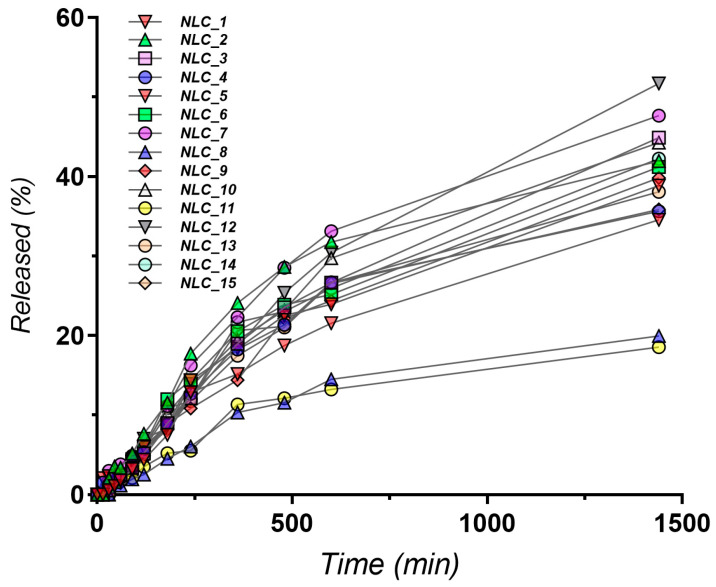
Comparative in vitro release profiles for the experimental NLC formulations.

The amount of docetaxel released within the first 24 h exhibited significant variability, ranging from 19.93% to 51.71% (Figure 5), highlighting the substantial influence of formulation parameters on drug release kinetics. 

The regression model for 24 h release reveals a complex interplay between multiple factors. The significant positive coefficient for the interaction term between liquid lipids and docetaxel (5.57*BC) suggests a synergistic effect on drug release. This could indicate that higher concentrations of both components lead to structural changes within the NLCs, facilitating faster drug diffusion or erosion of the lipid matrix. Conversely, the negative coefficient for docetaxel concentration (−5.14*C) implies that increasing drug loading alone may hinder release, potentially due to increased drug–lipid interactions or a denser packing of docetaxel within the NLC core.

To determine the optimal formulation based on the desired attributes—minimizing particle size, narrow particle size distribution (express as minimum PDI), and minimum zeta potential (since the particles are negatively charged) while maximizing 24 h drug release—a desirability function was employed. This approach involved transforming each response into a dimensionless desirability value (ranging from 0 to 1) and then calculating an overall desirability score. A desirability value close to 1 indicates a formulation closely aligned with the desired characteristics.

**Figure 6:**
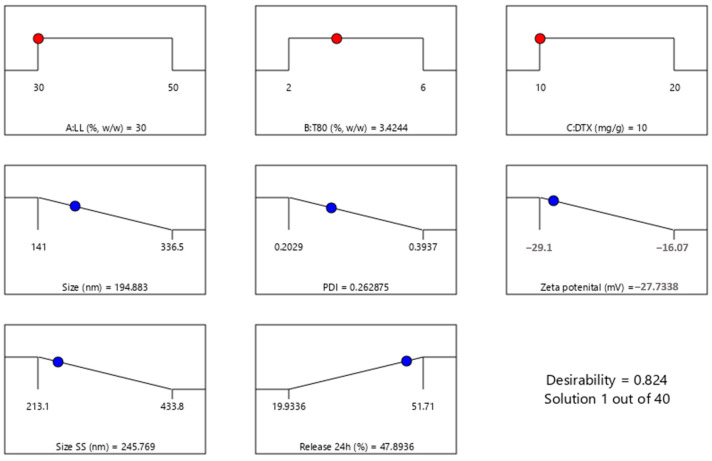
Optimization of the NLC formulation, based on the maximum value for the desirability function, highlighting the variables leading to the maximum desirability (in red) and the respective predicted responses (in blue).

The analysis yielded a desirability score of 0.824 for the formulation containing 30% liquid lipids (A), 3.42% Tween 80 (B), and 10 mg/g docetaxel (C), respectively (Figure 6). 

**Figure 7:**
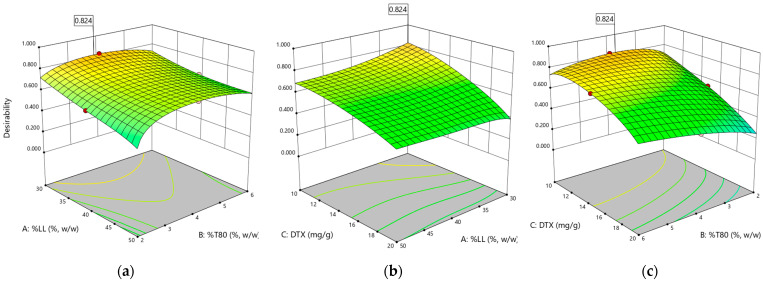
3D surface plots for desirability function analysis, depicting the combined effects of (**a**) liquid lipid (A) and surfactant concentration (B), (**b**) liquid lipid (A) and docetaxel (C), and (**c**) surfactant (B) and docetaxel (C) on desirability.

Figure 7 visually represents the 3D surface plots for the desirability function analysis, illustrating the influence of the formulation variables on the overall desirability score. This graphical representation aids in understanding the trade-offs and compromises inherent in optimizing multiple responses simultaneously. 

Guided by the desirability function analysis, the optimal formulation consisting of 30% liquid lipid, 3.5% Tween 80, and a docetaxel loading of 10 mg per gram of lipids was selected for further investigation. To validate the reproducibility of the formulation process and confirm the predicted characteristics, both the optimized docetaxel-loaded NLCs and a control formulation (devoid of docetaxel) were prepared in triplicate. These formulations underwent further characterization to assess their physicochemical properties and confirm the robustness of the optimized formulation. 

### 3.4. Characterization of the Optimized NLC Formulation

#### 3.4.1. Size, PDI, Zeta Potential, and Entrapment Efficiency

The optimized formulations (NLC-DTX) and the control counterpart (NLC-Blank), prepared without DTX, underwent characterization, and their physicochemical properties are detailed in Table 4.

While the inclusion of DTX resulted in a marginal enlargement of the nanoparticles, the minimal PDI values (<0.3) exhibited by both types of samples suggest a uniform size distribution, both immediately after preparation and after 30 days [78,82]. Additionally, the zeta potential values displayed notable deviations from neutrality, aligning with the sought-after attribute for pharmaceutical formulations that target enduring stability [13,83,84,85]. Worth mentioning is the superior loading capacity of the optimized formulation in comparison to analogous lipid-based nanoparticles, in accordance with findings from previous literature sources [86,87,88]. 

#### 3.4.2. In Vitro Release Kinetics

**Figure 8:**
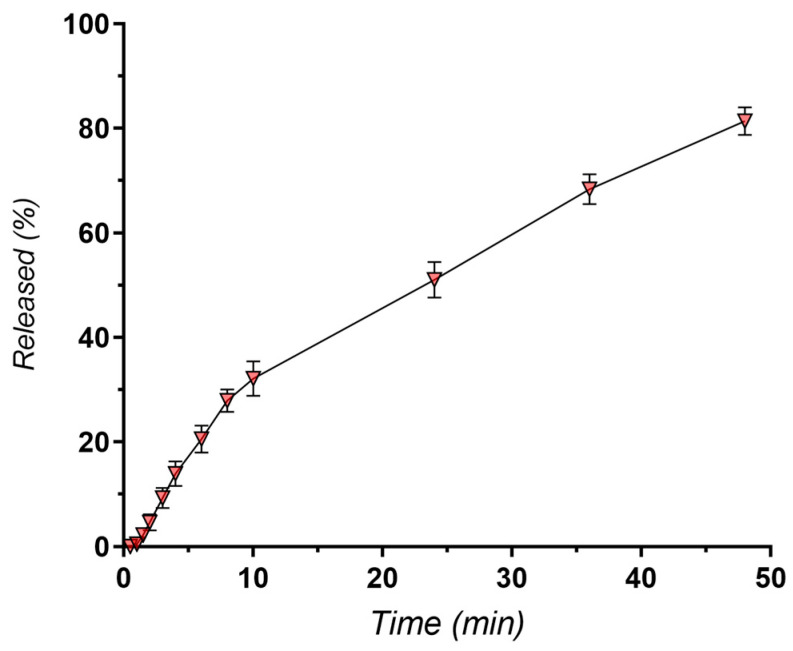
In vitro release profile for encapsulated docetaxel determined at 37 °C, receptor medium composed of PBS: ethanol (80:20, *v*/*v*), *n* = 6.

**Figure 9:**
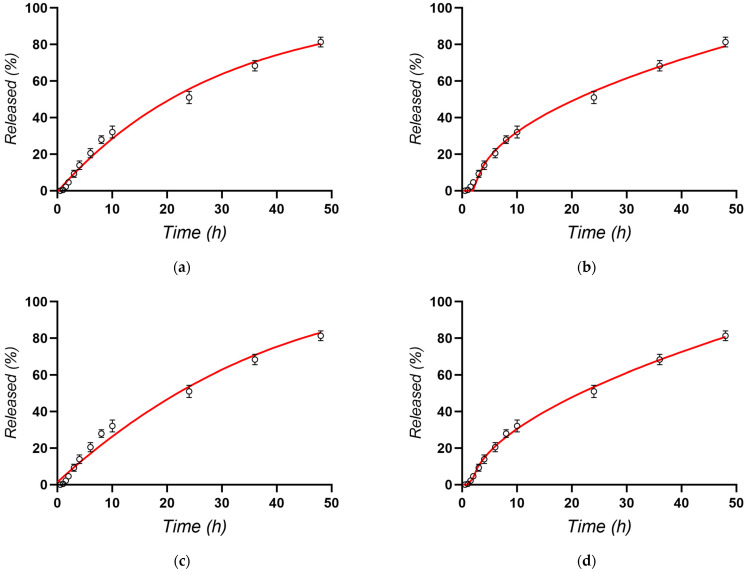
In vitro release kinetics (**a**) first order model, (**b**) Higuchi model, (**c**) Hixson–Crowell model, and (**d**) Korsmeyer–Peppas model.

Initial release studies across all experimental formulations revealed that only up to 50% of the incorporated docetaxel was released in 24 h, indicating a sustained release profile. To gain a more complete understanding of the release behavior, particularly for the optimized formulation, the release experiment was extended to 48 h. This extended timeframe allows for a more accurate assessment of the release mechanism and provides valuable insights into the potential of the formulation to achieve prolonged drug delivery to the tumor site. Figure 8 illustrates the in vitro release kinetics of docetaxel from the optimized nanostructured lipid carrier (NLC) formulation. Notably, approximately 50% of the encapsulated docetaxel was released within the first 24 h, and approximately 80% was released within 48 h (Figure 8). To gain a deeper understanding of the underlying mechanisms governing this release behavior, the experimental data were fitted to various mathematical models, such as Higuchi, first-order, Korsmeyer–Peppas, or Hixon–Crowell (Figure 9) [88,89,90]. 

Table 5 provides an overview of the parameters derived from the mathematical models employed to describe the release profiles of docetaxel from NLC-DTX.

The results indicate that the Korsmeyer–Peppas model, with the highest regression coefficient (R² = 0.9971), provides the best fit for describing docetaxel release from NLC formulations. The release exponent (*n* = 0.564) suggests that the release mechanism is primarily diffusive, adhering to Fick’s law, which is time-dependent on the square root of time. The strong correlation with the Higuchi model further supports the significant role of Fickian diffusion, where drug release is proportional to the square root of time [91,92,93]. This suggests that docetaxel release is primarily governed by its diffusion through the lipid matrix of the NLCs. However, the n value slightly exceeding 0.5 in the Korsmeyer–Peppas model also implies a contribution from erosion-based release mechanisms, indicating a complex interplay of diffusion with minor erosion processes [94,95].

#### 3.4.3. Scanning Electron Microscopy (SEM)

**Figure 10:**
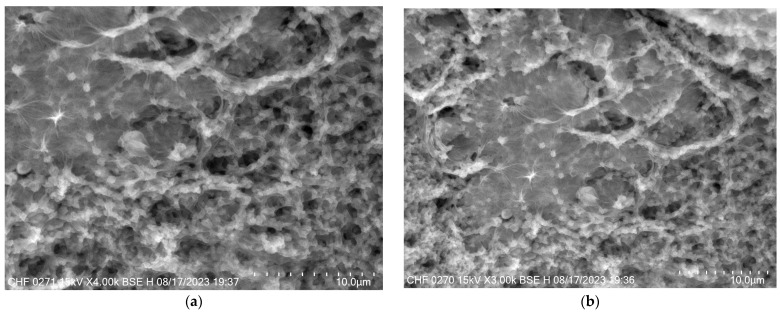
SEM micrographs of the optimized formulations: (**a**) NLC-Blank, (**b**) NLC-DTX.

SEM images provide valuable insights into the morphology of the nanostructured lipid carriers. Both formulations—with and without docetaxel—displayed uniformly sized particles (Figure 10). Importantly, the inclusion of docetaxel in the formulation did not affect the morphology of the nanoparticles, indicating that the presence of the API does not disrupt the structural integrity or uniformity of the carriers. This observation underscores the compatibility of docetaxel with the lipid matrix and the robustness of the NLC formulation process. There was also observed irregular form of NLCs into the SEM images which can be attributed to drying artifacts.

#### 3.4.4. NLC Nanostructure Evaluation through Combined XRD and Cryo-TEM Analysis

**Figure 11:**
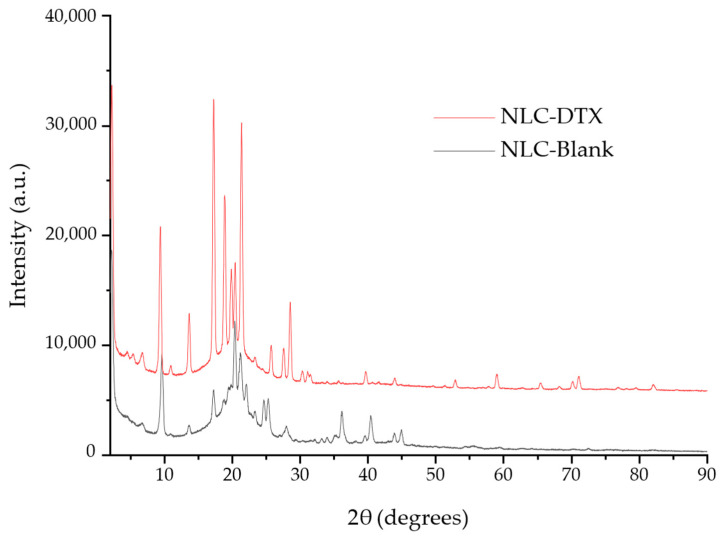
X-ray diffractograms recorded for NLC-Blank and NLC-DTX samples.

A combination of XRD and cryo-TEM was used to investigate the nanostructure of the obtained NLCs. XRD analysis (Figure 11) was performed on both the unloaded NLC formulation (NLC-Blank) and on the DTX-loaded carriers (NLC-DTX).

The XRD data for both NLC-Blank and NLC-DTX display numerous peaks, each corresponding to specific 2θ angles and d-spacings. The presence of multiple peaks at distinct d-spacings indicates crystalline structures with ordered atomic arrangements [96].

The strong, sharp peak observed in diffractograms at a low angle for both NLC-Blank and NLC-CTX formulations (2θ = 2.21°) is a good indication of long-range order in the NLCs, and it is frequently associated with lamellar or hexagonal phases. The very strong first peak, followed by weaker peaks at roughly multiples of its position, align with the expected features of lamellar phases. The first peak represents the primary d-spacing between layers, and the subsequent peaks are higher-order reflections [97].

The first four peaks show a clear 1:2:3:4 relationship in their d-spacings (39.91:19.37:13.14:9.16 Å), which is a strong indicator of a lamellar structure. This suggests a repeating layered structure with a basal plane spacing of approximately 39.9 Å. 

The presence of multiple peaks in the higher-angle region, especially the cluster between 17 and 25 degrees, further supports a lamellar structure. These peaks likely represent reflections from planes with smaller d-spacings within the layered arrangement [98].

The XRD analysis of NLC-DTX reveals several peaks specific to Docetaxel’s crystalline form at (°, 2ϴ): 17.25 (0,2,4), 18.87 (1,2,3), 19.82 (0,1,8), 20.39 (1,1,7), 21.34 (2,0,3), 25.67 (1,2,8), 27.62 (2,0,8), 28.48 (2,1,8), 30.27 (2,3,2), 31.05 (1,4,1), 31.45 (0,2,12), 52.82 (5,1,3), 58.95 (5,1,11), 65.35 (1,7,15), 68.14 (0,4,25), 70.09 (4,4,19), 70.99 (2,3,26), 76.82 (1,9,12), 79.51 (4,2,26), and 82.16 (1,9,16). Assignment of the diffraction peaks was performed by comparison with ICDD PDF Card No. 02-100-0701. The presence of these peaks can be interpreted as evidence of the partial crystallinity of Docetaxel within the lipid carrier matrix.

**Figure 12:**
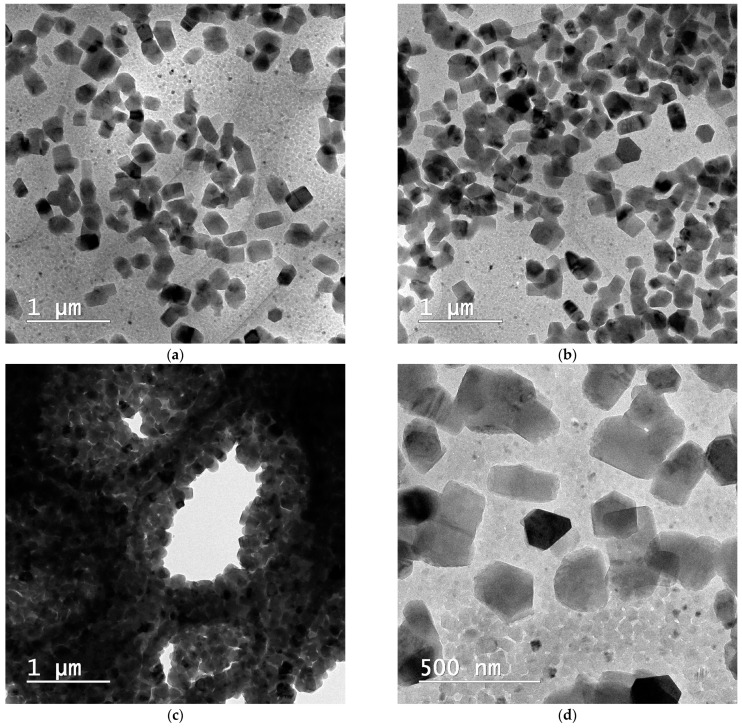

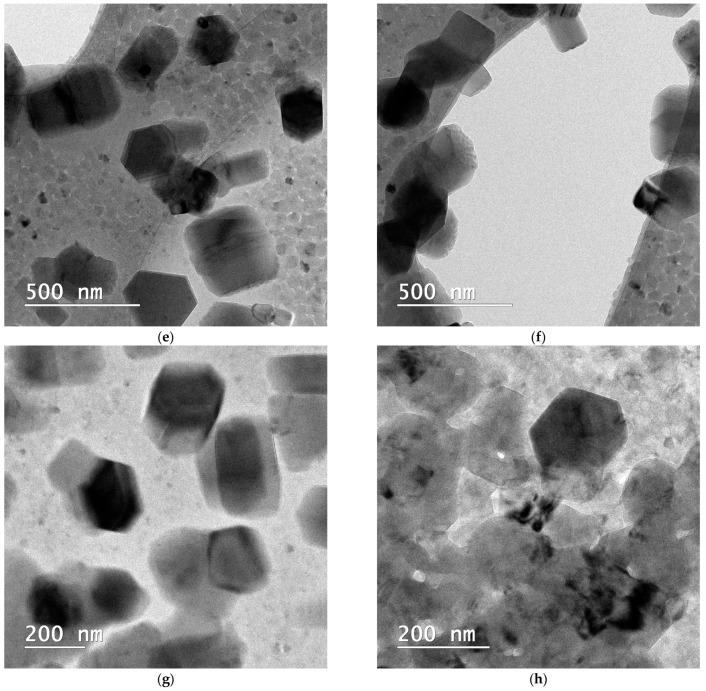
Cryo-TEM micrographs of the optimized formulations: (**a**) NLC-Blank (5000×), (**b**) NLC-DTX (5000×), (**c**) network of densely packed NLC-DTX (5000×), (**d**) NLC-Blank (14,500×), (**e**) NLC-DTX (14,500×), (**f**) NLC-DTX (14,500×) with lamellar ordered organization, (**g**) NLC-Blank (29,000×), and (**h**) NLC-DTX (29,000×).

The observed lamellar structures in the XRD data align with the layered arrangements seen in the cryo-TEM images (Figure 12f). This supports the hypothesis that the nanoparticles possess a lamellar internal structure.

The cryo-TEM images reveal key aspects of particle size, shape, and distribution, highlighting the morphology of the NLCs at different magnifications (Figure 12). Cryo-TEM images confirm the particle size of approximately 200 nm for the nanoparticles. By comparing the NLC-Blank and NLC-DTX formulations, it can be observed that the entrapment of docetaxel in nanoparticles does not modify their shape or size. 

The TEM images at lower magnifications (5000×) exhibit a well-dispersed system of nanoparticles (Figure 12a–c). The particles appear relatively uniform in shape and size, indicating a homogeneous formulation. Even when densely packed (Figure 12c), clear voids or pores are visible between the particles, indicating the absence of significant aggregation, and suggesting that the NLCs are stable and maintain their integrity during the preparation and imaging processes, which is crucial for consistent drug delivery and bioavailability.

Higher magnification images (11,500×, 14,500× and 29,000×) provide a closer look at individual nanoparticles, revealing overall well-defined and consistent shapes. The presence of elongated shapes with sharp edges indicates that these particles may be composed of stacked layers of parallel lamellae.

These assumptions are further confirmed by the lamellar packing into a hexagonal arrangement at a larger scale, as observed in Figure 12f. This lamellar packing within the hexagonal particles is expected to provide a stable structure that can offer controlled and sustained drug release, potentially due to increased surface area as well as enhanced stability [99,100]. This arrangement is further confirmed by the overall morphology of the particles, which appear elongated with sharp edges longitudinally, due to the parallel packing of the lamellar systems, and hexagonal transversally. Interestingly, skin lipids are also known for their capacity to form lamellar structures, further underlying the biomimetic and biocompatible nature of the prepared NLCs [101]. To the best of our knowledge, this is the first report demonstrating lamellar packing within hexagonal particles for docetaxel NLC formulations.

The combined XRD and TEM data suggest that the lipid matrix appears to form lamellar arrangements, while docetaxel is present in both crystalline and amorphous states. This mixed structural arrangement can be beneficial for drug delivery, providing a balance between stability and controlled drug release. 

In conclusion, the TEM and XRD analyses collectively demonstrate that the NLC formulations exhibit desirable structural characteristics, including uniform particle size, crystallinity, and potential lamellar arrangements. Their features are indicative of a stable and effective drug delivery system, capable of providing controlled and sustained release of docetaxel. This comprehensive analysis supports the potential use of these NLCs in clinical applications for cancer therapy.

#### 3.4.5. Thermal Analysis

Table 6 displays the temperature intervals and the associated mass loss for each excipient included in the NLC matrix. 

DTX is stable up to approximately 120 °C. Up to approximately 120 °C, it exhibits a mass loss of 0.35%, representing incorporated water. Subsequently, it decomposes in two main stages: the first between approximately 120 °C and 320 °C, with a mass loss of 56.48%, and the second between approximately 320 °C and 560 °C, with a mass loss of 26.70%. At the end of the analysis, a 16.53% residue was found. Additionally, the decomposition is characterized by two main peaks in derived weight (%/min), at 205 °C and 343 °C, respectively. Both the blank formulation (NLC-Blank) and the formulation containing DTX (NLC-DTX) exhibit similar thermal behaviour, each showing four stages: the first one with a loss of 0.57% up to approximately 100 °C representing the water content, the second one between approximately 100 °C and 220 °C with a mass loss of 6.40% for blank formulation, respectively, of 6.27% for NLC-DTX. The third continued until approximately 320 °C with a mass loss of 49.67%, for NLC-Blank and 51.32% for NLC-DTX. In the last stage up to 700 °C, the blank formulation lost 43.09% and the NLC with DTX entrapped 41.41%. Both decompositions of the formulations are characterized by three main peaks in derived weight (%/min). For NLC-Blank they are presented at 219 °C, 309 °C, and 390 °C, while NLC-DTX displays these peaks at 220 °C, 319 °C, and 390 °C.

**Figure 13:**
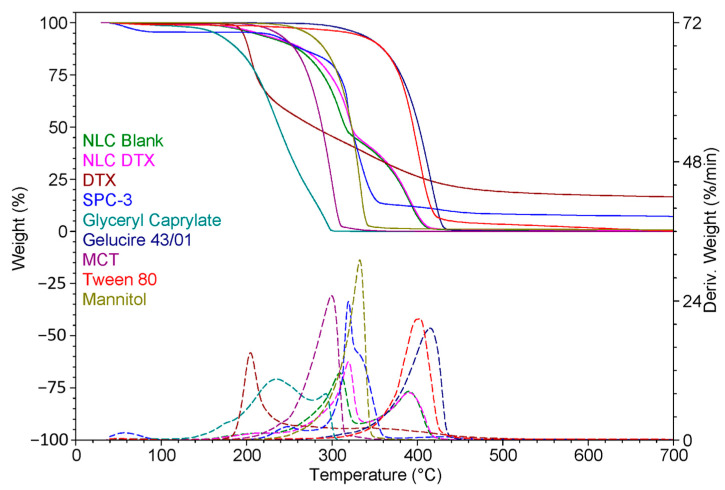
Thermal Analysis of NLC-DTX formulation TGA curves showing the weight loss (top) and derivative weight loss (bottom) as a function of temperature for various components of the NLC-DTX formulation.

Figure 13 shows the results of the TGA analysis, comparing NLC-Blank, NLC-DTX, DTX, and excipients, which highlights the differences in thermal behaviour among the samples.

### 3.5. In Vitro Biological Testing 

Cancer progression is driven by the delicate balance between uncontrolled cell proliferation and the inhibition of the apoptotic process. While healthy cells undergo apoptosis to eliminate damaged, useless, or potentially harmful cells [102], tumor cells often circumvent this process, enabling unchecked cell proliferation. Furthermore, dysregulation of the cell cycle, a tightly controlled sequence of growth and division, significantly contributes to tumor development. Thus, targeting these aberrant processes is a crucial strategy in cancer therapy. 

The cell cycle is another process implicated in tumor progression. It comprises a complex series of events, including cellular growth, DNA replication, and division. The cell cycle is divided into distinct phases—G1, S, G2, and M—which are regulated by various checkpoints. These checkpoints enable the cell to assess signals from its internal and external environment, ensuring favorable conditions for progression to the next phase. However, if these regulatory mechanisms malfunction, cells can divide uncontrollably, leading to tumor formation [103,104].

Our study aimed to investigate the effect the NLC-DTX formulation on the SK-MEL-24-line (melanoma) tumor cells compared to HUVEC line normal cells. We analyzed the compounds’ impact on cell proliferation, apoptosis, and cycle phases. The positive control of the study was the cytostatic, most commonly used in treating melanomas. The cell lines were exposed to various concentrations of the compounds for 24 and 48 h.

In order to determine the apoptotic process, we used double labeling with annexin V-FITC/PI and flow cytometry. By doing so, we could quantify the population of cells in early or late apoptosis, as well as those in necrosis. Additionally, we compared the effects of analyzed compounds on tumor cells to those on normal cells.

The cell cycle analysis on tumor or normal cells treated with the compounds was also performed using flow cytometry. This technique allowed us to analyze the amount of DNA present in the cells and the distribution of the phases of the cell cycle. The resulting data was then presented as a graph depicting the levels of PI staining (a dye used to visualize DNA) in the cells.

#### 3.5.1. Cell Viability Assay

**Figure 14:**
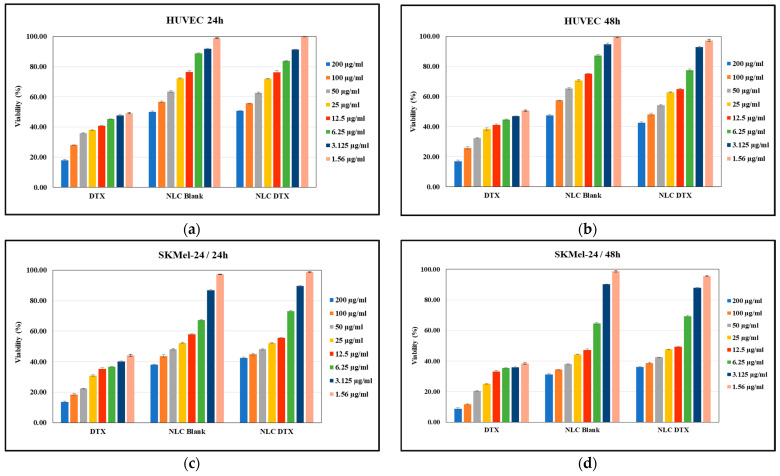
The effects of the analyzed compounds on cell viability: (**a**) HUVEC normal cells for 24 h, (**b**) HUVEC normal cells for 48 h, (**c**) SK-MEL-24 tumoral cells for 24 h, and (**d**) SK-MEL-24 tumoral cells for 48 h.

The effects of the analyzed formulations (NLC-DTX, NLC-Blank) versus docetaxel solution (DTX) on HUVEC normal cell viability are related in Figure 14a,b, while their effect on SK-MEL-24 tumoral cell viability are presented in Figure 14c,d. In both cases, the cells were treated with formulations in the range of concentrations of 1.56–200 μg/mL for 24 h and for 48 h, respectively. The obtained dose–effect curves were used to illustrate the correlation between concentration and its impact on cell viability. Untreated cells were considered to have 100% viability.

As expected, data presented in Figure 14 demonstrate that cell viability, in both tumor and normal cells, is concentration dependent. This dose–response relationship is crucial for determining the optimal therapeutic window—a dose that maximizes anti-tumor activity while minimizing side effects on healthy tissues.

Free DTX exhibited significant toxicity towards both SK-MEL-24 melanoma cells (Figure 14c,d) and HUVEC normal cells (Figure 14a,b). This indiscriminate cytotoxicity, a hallmark of conventional chemotherapy, was evident by the substantial reduction in HUVEC viability (over 50% cell death) at all tested concentrations after 24 and 48 h.

Importantly, encapsulating DTX within NLCs significantly mitigated its toxicity on normal cells. The IC50 of NLC-DTX on HUVECs was higher than the highest tested concentration (200 μg/mL), indicating a substantial increase in safety compared to free DTX (IC50 < 1.56 μg/mL). At concentrations below 50 μg/mL, NLC-DTX showed comparable effects to NLC-Blank on HUVEC viability, suggesting a protective effect of the NLC delivery system. 

Despite this improved safety profile, NLC-DTX retained potent anti-tumor activity against SK-MEL-24 cells. Treatment with NLC-DTX at concentrations between 12.5 and 50 μg/mL reduced SK-MEL-24 cell viability by 50% after 24 h, with the inhibitory effect sustained and slightly enhanced after 48 h (calculated IC50 value of 11.6 μg/mL at 48 h). This highlights the potential of NLC-DTX in effectively targeting and inhibiting melanoma cells while potentially minimizing adverse effects on normal cells.

For NLC-Blank a cytotoxic effect was also observed at concentrations higher than 125 μg/mL, suggesting an inherent toxicity of the NLC carrier itself.

The data presented in Figure 14a,b show that the NLC-DTX formulation is less toxic to HUVEC normal cells compared to DTX, while maintaining cytotoxic activity on the SK-MEL-24 cells. This finding is promising for developing strategies to mitigate the adverse effects of chemotherapy on normal tissues while maintaining its efficacy against tumors. 

In summary, our study provides valuable insights into the concentration-dependent effects of NLC-DTX formulations on both tumor and normal cells. These findings contribute to optimizing therapeutic doses and developing strategies to reduce side effects on normal cells while enhancing the efficacy of chemotherapy against melanoma and potentially other cancers.

#### 3.5.2. Apoptotic Process Induced by the Analyzed Formulations

The apoptotic process in tumor cells is significantly inhibited, which ensures cell proliferation and the evolution of the tumor process. The analysis of the effects of the studied formulations on the apoptotic process of cells lead to the selection of the formulations capable of inducing apoptosis in tumor cells without significantly affecting the apoptotic process of normal cells. Thus, the study analyzed how certain substances influence the apoptotic process to identify formulations with therapeutic potential by selectively inducing apoptosis in SK-MEL-24 cancer cells. The effects caused by the studied compounds on the apoptotic process in normal or tumor cells were analyzed using the flow cytometry technique.

**Figure 15:**
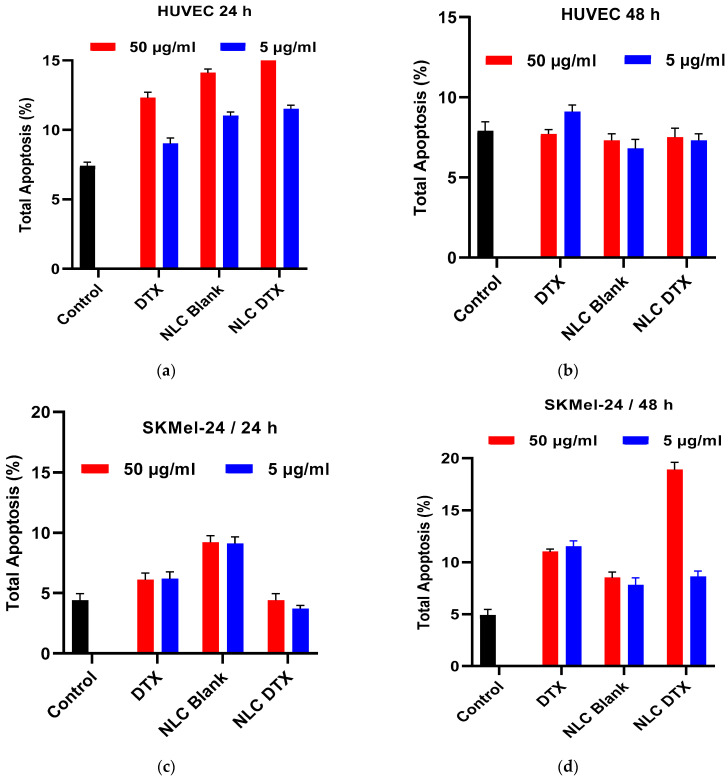
The effects of formulations at concentrations of 5 and 50 μg/mL compared to untreated controls on the apoptotic process of: (**a**) HUVEC normal cells at 24 h, (**b**) HUVEC normal cells at 48 h, (**c**) SK-MEL-24 tumoral cells at 24 h, and (**d**) SK-MEL-24 tumoral cells at 48 h.

To investigate the selective cytotoxic effects of NLC-DTX, we analyzed its impact on apoptosis in both SK-MEL-24 tumor cells and normal HUVEC cells. Two reference concentrations (5 and 50 μg/mL) were chosen based on cell viability data, and treatments were conducted for 24 and 48 h (Figure 15).

Untreated HUVEC cells exhibited a low basal level of apoptosis, approximately 7–8%, regardless of the incubation time (Figure 15a,b). Treatment with NLC-Blank or DTX for 24 h led to a slight increase in apoptosis compared to untreated controls, up to 14.1% for NLC-Blank and 12.3% for DTX at 50 μg/mL (Figure 15a). Similarly, NLC-DTX induced a modest increase in apoptosis at 24 h, without registering an additive effect of the two components (NLC-Blank and DTX). Importantly, the apoptotic effects of all treatments on HUVEC cells were transient, disappearing after 48 h (Figure 15b).

Untreated SK-MEL-24 cells also displayed a low percentage of apoptotic cells (approximately 4–5%) at both time points (Figure 15c,d). In contrast to the transient effects observed in HUVEC cells, treatment with DTX, NLC-Blank, and NLC-DTX all led to sustained increases in apoptosis in SK-MEL-24 cells.

DTX treatment (5 and 50 μg/mL) resulted in a two-fold increase in apoptosis after 48 h. 

NLC-Blank (5 and 50 μg/mL) induced a 1.5-fold increase in apoptosis at both 24 and 48 h, suggesting an inherent pro-apoptotic effect of the NLC carrier itself on the SK-MEL-24 tumor cell line. This increase in apoptosis can be attributed to the fact that the lipid composition of the NLCs may disrupt cellular membrane integrity, as components like Gelucire^®^ 43/01 and MCT can destabilize lipid bilayers. Secondly, the presence of nanoparticles can generate reactive oxygen species (ROS), causing oxidative stress. Thirdly, the carrier system itself may exhibit inherent cytotoxicity, contributing to the pro-apoptotic effect. Finally, enhanced cellular uptake of nanoparticles by tumor cells, may result in higher intracellular concentrations and amplified effects.

These combined effects—lipid composition disruption, ROS generation, inherent cytotoxicity, and enhanced uptake—explain the increased apoptosis observed with NLC-Blank treatment, highlighting the importance of considering nanoparticle properties in biological evaluations.

NLC-Blank (5 and 50 μg/mL) induced a 1.5-fold increase in apoptosis at both 24 and 48 h, suggesting an inherent pro-apoptotic effect of the NLC carrier itself on this tumor cell line, likely due to a combination of lipid composition disrupting membrane integrity, nanoparticle-induced generation of reactive oxygen species (ROS), which can lead to oxidative stress, inherent carrier cytotoxicity, and enhanced cellular uptake. 

Notably, NLC-DTX exhibited the most potent pro-apoptotic effect on SK-MEL-24 cells. At the higher concentration (50 μg/mL), NLC-DTX induced a 3.8-fold increase in apoptosis after 48 h. Even at the lower concentration (5 μg/mL), NLC-DTX doubled the percentage of apoptotic SK-MEL-24 cells after 48 h.

These findings demonstrate the superior efficacy of NLC-DTX in inducing apoptosis specifically in SK-MEL-24 tumor cells while sparing normal HUVEC cells. The enhanced pro-apoptotic effect of NLC-DTX compared to free DTX or NLC-Blank suggests a synergistic effect of the NLC delivery system and the encapsulated drug. This selective cytotoxicity underscores the potential of NLC-DTX as a targeted cancer therapy, capable of maximizing antitumor effects while minimizing off-target toxicity.

#### 3.5.3. Cell Cycle Phases

Monitoring cell cycle dynamics and understanding the underlying molecular mechanisms are essential for gaining insights into cellular behavior, especially in the tumor process. The S phase of the cell cycle is a critical stage during which DNA replication occurs. It is the phase where the cell duplicates its DNA in preparation for cell division. The G2 phase follows the S phase and mitosis (M phase). The analysis of cell cycle phases provides information that helps to understand the dynamics and characteristics of cell division [105]. 

Staining cells with a fluorescent dye that specifically binds to DNA allows visualization and quantification of DNA content in individual cells by flow cytometry. The distribution of DNA content obtained from flow cytometry reflects the different phases of the cell cycle, G1, S, and G2+M. By comparing the relative proportions of cells in each phase, information about cell cycle dynamics can be obtained. Flow cytometric analysis provides information on cell proliferation rates, the duration of each phase, and overall progression through the cell cycle. The changes in the cell cycle profile show how these analyzed compounds influence the cell division process.

**Figure 16:**
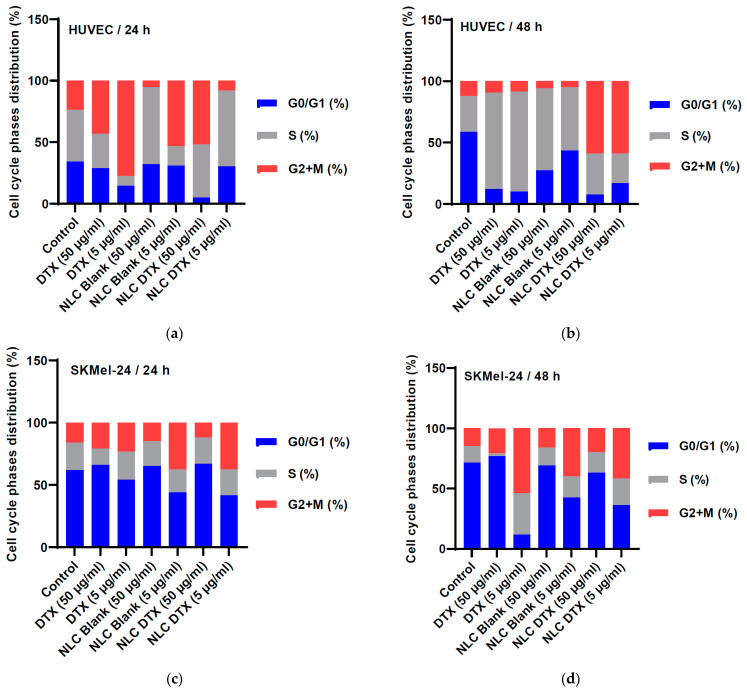
The influence of the analyzed formulations on the cell cycle in (**a**) normal cells HUVEC after a 24 h treatment, (**b**) normal cells HUVEC after a 48 h treatment, (**c**) tumoral cells SK-MEL-24 after a 24 h treatment, and (**d**) tumoral cells SK-MEL-24 after a 48 h treatment.

To understand the mechanisms underlying the observed effects on cell viability and apoptosis, we investigated the influence of DTX, NLC-Blank, and NLC-DTX on cell cycle progression in both HUVEC and SK-MEL-24 cells using flow cytometry (Figure 16).

Treatment with DTX and NLC-Blank led to distinct cell cycle alterations in HUVEC cells (Figure 16a,b). Thus, DTX at 24 h causes a decrease in the S phase accompanied by an increase in the G2 phase, followed by an increase in the S phase and a significant decrease in the G1 phase, independent of the concentration, at 48 h.

DTX (50 μg/mL) induced a G2/M arrest at 24 h, followed by a decrease in the G1 population, and an increase in the S phase at 48 h. Conversely, NLC-Blank (50 μg/mL) increases the G2 phase and increases the S phase at 24 h and 48 h. NLC-Blank 5 μg/mL at 24 h reduces the S phase and increases the G2 phase, and at 48 h, induces an increase in the S phase and a decrease in the G2 phase (Figure 16). These findings suggest that DTX and the NLC carrier alone may influence cell cycle progression through different mechanisms.

Interestingly, NLC-DTX (50 μg/mL) elicited cell cycle effects similar to DTX, by inducing an increase in the G2 phase at 24 h and a decrease of the G1 phase at 48 h. 

However, at a lower concentration (5 μg/mL), NLC-DTX displayed a distinct profile, inducing a significant decrease in the G2 phase accompanied by an increase in the S phase at 24 h, followed by a decrease in both G1 and S phases and a significant increase in the G2 phase at 48 h. This suggests a potential concentration-dependent effect of NLC-DTX on cell cycle dynamics in HUVEC cells.

In SK-MEL-24 tumor cells, DTX, NLC-Blank, and NLC-DTX also exhibited distinct effects on cell cycle distribution (Figure 16c,d). DTX (50 μg/mL) at 24 h causes a decrease in the S phase accompanied by an increase in the G2 phase, while at 48 h, there was a significant decrease in the S phase. At a lower concentration (5 μg/mL), DTX decreases the G1 phase and increases the G2 phase regardless of the time treatment was applied. NLC-Blank (50 μg/mL) did not significantly alter cell cycle distribution in SK-MEL-24 cells. 

However, at a lower concentration (5 μg/mL), NLC-Blank at 24 h decreases the S phase and increases the G2 phase, and at 48 h induces a decrease in the G1 phase accompanied by an increase in the G2 phase.

The use of NLC-DTX (50 μg/mL) does not significantly change the cell cycle phases of SK-MEL-24 tumor cells, regardless of the duration of treatment. Using a lower concentration of NLC-DTX (5 μg/mL) led to an increase in the G2 phase of the cell cycle of SKMel-24 tumor cells at 24 h and 48 h (Figure 16c,d).

The results show that using the analyzed formulations and DTX in treating SK-MEL-24 tumor cells can act on the phases of the cell cycle in a different way. Thus, the recorded changes could involve the delay of the entry into the S phase or the extension of the time spent by the cells in G1 or G2. Also, slowing the cell cycle can allow more time for cellular processes, such as DNA repair, before cells engage in division.

DTX and each formulation evaluated induces a cell cycle arrest of SK-MEL-24 in a different phase, in which cells are temporarily stopped. The observed variations in cell cycle distribution suggest that these compounds may act through distinct mechanisms to influence cell cycle checkpoints and ultimately impact cell proliferation. The ability of NLC-DTX to modulate cell cycle dynamics, even at lower concentrations, highlights its potential as an effective therapeutic approach for skin cancer treatment.

These results open new research directions for studying the cellular mechanisms targeted by the analyzed compounds to optimize therapeutic strategies for cancer treatment. Understanding how compounds influence cell cycle dynamics can inform the development of more effective and targeted therapies while minimizing adverse effects on normal cells, thus advancing precision medicine approaches in cancer treatment.

### 3.6. Clinical and Industrial Translation Aspects

The development of docetaxel-loaded nanostructured lipid carriers (NLCs) for topical application represents a groundbreaking advancement in the therapeutic management of skin cancer. This innovative formulation aims to optimize the localized delivery of docetaxel, ensuring high drug concentrations at the tumor site while minimizing systemic exposure and associated adverse effects. Topical administration of NLC-DTX offers several notable clinical benefits, such as localized treatment, enhanced drug penetration, sustained release, and reduced side effects. 

Applying the NLC formulation directly to the skin concentrates docetaxel in the tumor region, enhancing its therapeutic efficacy and minimizing systemic exposure. This targeted approach reduces the risk of systemic toxicity, a common drawback of conventional chemotherapy, leading to a more favorable side effect profile for patients.

The nano-sized particles of the NLCs are designed to penetrate the stratum corneum, a significant barrier to topical drug delivery. This enhanced penetration capability ensures that adequate drug levels reach the dermal and subdermal layers, where many skin cancers are located. This targeted delivery translates to improved treatment efficacy and potentially higher rates of tumor regression. NLCs provide controlled and sustained release of docetaxel, maintaining therapeutic drug levels over extended periods. This sustained release not only improves treatment efficacy but also reduces the frequency of application needed, leading to improved patient compliance and convenience.

Confining docetaxel’s action to the application site significantly lowers the risk of systemic side effects such as immunosuppression, hair loss, and nausea, which are frequently observed with conventional chemotherapy. This localized action is particularly beneficial for elderly patients and those with comorbid conditions who are more susceptible to adverse effects from systemic treatments. The reduction in side effects contributes significantly to an improved quality of life for patients undergoing treatment.

In vitro cytotoxicity studies on human umbilical vein endothelial cells (HUVEC) and SK-MEL-24 skin cancer cells have yielded promising results, NLC-DTX formulations exerting selective cytotoxicity towards SK-MEL-24 cells, exhibiting a dose-dependent reduction in cell viability. This selective cytotoxicity suggests that the formulation effectively targets cancer cells while sparing normal cells, which is crucial for minimizing side effects. Compared to free docetaxel, the NLC-DTX formulation showed enhanced efficacy in reducing the viability of SK-MEL-24 cells. This increased efficacy can be attributed to the improved penetration and sustained release properties of the NLCs, which ensure prolonged exposure of cancer cells to the drug. The NLC-Blank (without docetaxel) exhibited minimal cytotoxicity towards HUVEC cells, indicating the biocompatibility of the carrier system. The favorable safety profile of the NLC formulation supports its potential for clinical use, minimizing the risk of adverse reactions in normal tissues.

The formulation’s ability to provide sustained and controlled drug release makes it an ideal candidate for combination therapy. It can be used alongside other topical agents to enhance therapeutic outcomes and potentially overcome drug resistance.

The formulation of NLC-DTX for topical use also presents several opportunities in terms of industrial transfer. The manufacturing processes used for creating NLCs, such as high-pressure homogenization and emulsification-ultrasonication, are highly scalable. These processes can be adapted for large-scale production, ensuring consistency, cost-effectiveness, and the ability to meet high market demand.

The stability studies have demonstrated that NLC-DTX formulations maintain their physicochemical properties and therapeutic efficacy over extended storage periods. This stability is crucial for ensuring that the product remains effective from the point of manufacture to the time of administration. Furthermore, topical formulations generally encounter fewer regulatory challenges compared to systemic treatments. The localized nature of the NLC-DTX treatment, coupled with reduced systemic exposure, may expedite regulatory approval processes, facilitating quicker market entry. Last but not least, topical treatments are often more acceptable to patients due to their non-invasive nature. The ease of application and reduced side effects associated with NLC-DTX are likely to enhance patient adherence to treatment regimens.

## 4. Conclusions

This study presents a comprehensive investigation into the development and characterization of docetaxel-loaded NLCs for topical skin cancer treatment. Utilizing Design of Experiments, we systematically optimized critical formulation parameters to achieve NLCs with superior performance characteristics. This approach, encompassing rigorous physicochemical characterization and promising in vitro cell line studies, positions these NLCs as strong candidates for further preclinical and clinical evaluation.

Our findings highlight the potential of this formulation to significantly improve therapeutic outcomes in skin cancer treatment. The clinical benefits of localized, sustained drug delivery, coupled with the industrial feasibility and positive in vitro results, underscore the potential of this formulation to significantly improve therapeutic outcomes. This novel approach has the potential to transform the current landscape of skin cancer treatment, offering a more effective and patient-friendly alternative to conventional chemotherapy. However, the translation of these in vitro findings to in vivo efficacy and safety profiles necessitates cautious consideration.

While further preclinical and clinical studies are essential to fully validate the efficacy and safety of NLC-DTX, this research provides a significant step towards more targeted and efficient skin cancer therapies. The synergy between systematic optimization through DoE and comprehensive analytical methods employed in this study contributes to advancing the field of nanomedicine for improved patient outcomes.

## Figures and Tables

**Table 1:** Experimental variables and response optimization criteria used in the experimental design.

Variables	Level		
	Low (−1)	Medium (0)	High (+1)
**LL** (**%** *w*/*w*)	30	40	50
**T80%**	2	4	6
**DTX** (**mg/g**)	10	15	20
**Responses**		**Optimization**	
**Size** (**nm**)		minimum	
**Size SS** (**nm**)		minimum	
**PDI**		minimum	
**ZP** (**mV**)		maximum (in absolute value)	
**Release 24 h** (**%**)		maximum	

**Table 2:** Results from the Box–Behnken design for the optimization of docetaxel NLCs, depicting both the independent (LL, T80, and DTX) and dependent variables (Size, PDI, Size SS, ZP, and Release 24 h). The independent variables are also denoted by capital letters: A = LL, B = T80, C = DTX.

Variables				Responses				
Formulation	A: LL (%, *w*/*w*)	B: T80 (% *w*/*w*)	C: DTX (mg/g)	Size (nm)	PDI	Size SS (nm)	ZP (mV)	Release 24 h (%)
NLC_1	30	4	20	141	0.2993	434	−20.68	34.54
NLC_2	40	2	10	291	0.2083	239	−28.93	41.96
NLC_3	30	2	15	287	0.2189	328	−29.01	39.21
NLC_4	40	6	20	179	0.2804	310	−16.07	40.07
NLC_5	40	4	15	193	0.2162	282	−24.22	38.86
NLC_6	40	4	15	200	0.2174	267	−22.42	41.21
NLC_7	50	4	10	217	0.2873	226	−17.94	47.67
NLC_8	40	2	20	337	0.2987	334	−20.63	19.93
NLC_9	40	6	10	158	0.3937	248	−22.98	39.83
NLC_10	30	4	10	168	0.2463	270	−27.36	44.26
NLC_11	50	2	15	323	0.2029	286	−25.40	37.30
NLC_12	30	6	15	176	0.2276	300	−23.32	51.71
NLC_13	50	4	20	220	0.2843	213	−16.26	38.07
NLC_14	50	6	15	186	0.2041	242	−19.73	42.23
NLC_15	40	4	15	214	0.2155	268	−24.91	35.86

**Table 3:** ANOVA results for the response variables used in the optimization of docetaxel NLCs.

Dependent Variable	Source	Sum of Squares	df	Mean Square	F-Value	*p*-Value
Size (nm)	Model	48,977.18	3	16,325.73	51.00	<0.0001
	A-LL (%, *w*/*w*)	3806.28	1	3806.28	11.89	0.0054
	B-T80 (%, *w*/*w*)	36,301.65	1	36,301.65	113.41	<0.0001
	B²	8869.25	1	8869.25	27.71	0.0003
	Residual	3521.11	11	320.10	-	-
	Cor Total	52,498.29	14	-	-	-
PDI	Model	0.0341	4	0.0085	15.11	0.0003
	B-T80 (%, *w*/*w*)	0.0039	1	0.0039	6.95	0.0249
	C-DTX (mg/g)	0.0001	1	0.0001	0.1628	0.6951
	BC	0.0104	1	0.0104	18.40	0.0016
	C²	0.0197	1	0.0197	34.93	0.0001
	Residual	0.0056	10	0.0006		
	Cor Total	0.0397	14	-	-	-
Zeta potential (mV)	Model	214.82	4	53.71	19.93	<0.0001
	A-LL (%, *w*/*w*)	55.81	1	55.81	20.71	0.0011
	B-T80 (%, *w*/*w*)	60.28	1	60.28	22.37	0.0008
	C-DTX (mg/g)	69.44	1	69.44	25.77	0.0005
	C²	29.29	1	29.29	10.87	0.0081
	Residual	26.95	10	2.69	-	-
	Cor Total	241.77	14	-	-	-
Size SS (nm)	Model	36,456.67	3	12,152.22	23.28	<0.0001
	A-LL (%, *w*/*w*)	16,644.00	1	16,644.00	31.88	0.0001
	C-DTX (mg/g)	11,989.26	1	11,989.26	22.97	0.0006
	AC	7823.40	1	7823.40	14.99	0.0026
	Residual	5742.60	11	522.05		
	Cor Total	42,199.27	14	-	-	-
Released 24 h (%)	Model	590.00	5	118.00	11.37	0.0011
	A-LL (%, *w*/*w*)	2.48	1	2.48	0.2384	0.6370
	B-T80 (%, *w*/*w*)	157.02	1	157.02	15.13	0.0037
	C-DTX (mg/g)	211.19	1	211.19	20.34	0.0015
	BC	123.88	1	123.88	11.93	0.0072
	A²	95.43	1	95.43	9.19	0.0142
	Residual	93.43	9	10.38		
	Cor Total	683.42	14	-	-	-

**Table 4:** Physicochemical properties (size, PDI, size SS, PDI SS, EE %) of the NLC-DTX formulation selected by the Box–Behnken design and its control (*n* = 3, results depicted as mean ± SD).

Formulation	Size (nm)	PDI	Size SS (nm)	PDI SS	ZP (mV)	EE %	Release 24 h
NLC-DTX	199 ± 7.21	0.2793 ± 0.0139	277 ± 14.00	0.2734 ± 0.0220	−28.73 ± 3.55	99.16 ± 2.28	45.62 ± 3.19
NLC-Blank	187 ± 8.53	0.1955 ± 0.0379	327 ± 9.26	0.2327 ± 0.03646	−35.63 ± 3.70	-	-

**Table 5:** Docetaxel release parameters of the following different kinetics models.

Sample	First order		Higuchi		Hixson-Crowell		*Korsmeyer-Peppas*		
	R^2^	k_1_	R^2^	K_H_	R^2^	k_3_	R^2^	k_KP_	n
NLC-DTX	0.9890	0.034	0.9939	11.734	0.9814	0.009	0.9971	9.289	0.564

**Table 6:** Thermal decomposition of excipients.

Excipient	Temperature Interval(s) and Mass Loss				Residue
Gelucire 43/01 (G43/01)	270–450 °C				not present
	99.75%				
Glyceryl caprylate	50–120 °C	120–280 °C		280–330 °C	not present
	1.39%	81.96%		16.58%	
MCT	180–350 °C				not present
	99.97%				
S-PC-3	50–180 °C	180–270 °C	270–370 °C	370–540 °C	7.20%
	4.50%	9.19%	73.69%	4.50%	
Tween 80 (T80)	300–500 °C				not present
	95.51%				
Mannitol	220–500 °C				not present
	98.95%				

## Data Availability

The original contributions presented in the study are included in the article; further inquiries can be directed to the corresponding author.
